# Enhancer-mediated NR2F2 recruitment activates BGN to promote tumor growth and shape tumor microenvironment in papillary thyroid cancer

**DOI:** 10.7150/thno.113712

**Published:** 2026-01-01

**Authors:** Mei Tao, Xianhui Ruan, Jialong Yu, Qiman Dong, Wei Luo, Wei Zhang, Mengran Tian, Xiukun Hou, Linfei Hu, Jingzhu Zhao, Dapeng Li, Jie Hao, Songfeng Wei, Xiangqian Zheng, Ming Gao

**Affiliations:** 1 Department of Thyroid and Neck Tumor, Tianjin Medical University Cancer Institute and Hospital, National Clinical Research Center for Cancer, Key Laboratory of Cancer Prevention and Therapy, Tianjin's Clinical Research Center for Cancer, Tianjin, 300060, China.; 2 Department of Thyroid and Breast Surgery, Tianjin Key Laboratory of General Surgery in Construction, Tianjin Union Medical Center, Tianjin, 300121, China.

**Keywords:** BGN, Papillary thyroid cancer, enhancer-promoter loop, M2 macrophage, NR2F2

## Abstract

**Background:** Biglycan (BGN), a component of the extracellular matrix, has been closely associated with tumor progression. This study aimed to investigate the endocrine and paracrine roles of BGN in papillary thyroid carcinoma (PTC) to elucidate the underlying molecular mechanisms driving PTC development.

**Methods:** Multi-omics integration was used to assess BGN expression and its clinical relevance in PTC. Functional assays, including cell viability, colony formation, and cell cycle analysis, were used to evaluate the biological functions of BGN. RNA-seq identified key signaling pathways involved in BGN-mediated PTC progression. Immune cell infiltration and macrophage polarization were analyzed using single-cell RNA-seq and flow cytometry. CRISPR interference was employed to suppress BGN enhancer activity, while luciferase assays confirmed NR2F2's role in regulating BGN expression. The therapeutic potential of the NR2F2-specific inhibitor CIA1 was tested in PTC cell lines and mouse models.

**Results:** BGN overexpression in malignant PTC cells evaluated by BayesPrism deconvolution was linked to poor clinicopathological features in PTC. BGN knockdown inhibited cell proliferation and induced cell cycle arrest, which was rescued by recombinant BGN. Tumor-derived BGN activated the AKT signaling pathway, promoting tumor growth. Additionally, high BGN expression facilitated M2 macrophage polarization and immune evasion through TLR4 signaling. The NR2F2-BGN axis activated BGN transcription and AKT signaling. CIA1 treatment reduced BGN expression, suppressed cell proliferation, and modulated macrophage polarization.

**Conclusion:** Our findings highlight the NR2F2-BGN axis as a critical regulator of PTC progression. Targeting this axis offers a promising therapeutic strategy for PTC treatment and immune microenvironment modulation.

## Introduction

Biglycan (BGN), a class I small leucine-rich proteoglycan (SLRP), is an essential component of the extracellular matrix (ECM) and plays pivotal roles in tissue architecture, cell signaling, and immune regulation 1. As a structural ECM molecule, BGN contributes to tissue homeostasis and is broadly expressed in the immune, vascular, and musculoskeletal systems 2-4. Functionally, BGN can induce sterile inflammation by engaging Toll-like receptors (TLRs) and their co-receptors, thereby promoting tissue injury and pathological remodeling 5. Mounting evidence indicates that BGN is aberrantly overexpressed in both tumor and stromal compartments across multiple malignancies, where its elevated levels correlate with enhanced tumor aggressiveness and poor prognosis 6. Indeed, BGN upregulation has been reported in gastric, breast, bladder, lung, head and neck squamous cell carcinoma, esophageal, and ovarian cancers 7-14. Despite these observations, the transcriptional regulatory mechanisms underlying BGN expression in papillary thyroid carcinoma (PTC) and its functional significance within the tumor microenvironment (TME) remain poorly understood.

Epigenetic alterations have emerged as central drivers of thyroid carcinogenesis, impacting chromatin architecture, histone modifications, DNA methylation, and RNA modifications 15-17. These regulatory processes not only shape tumor cell biology but also influence immune cell infiltration and therapeutic response within the TME 18, 19. Recent studies highlight the interplay between epigenetics and tumor immunity, such as the modulation of immunotherapy efficacy by gut microbiota through epigenetic and metabolic crosstalk 20. In addition, stromal elements including cancer-associated fibroblasts (CAFs) and their extracellular vesicles act as powerful regulators of the TME, affecting immune evasion and treatment outcomes 21. Moreover, RNA modifications, such as N6-methyladenosine (m6A), have been linked to distinct TME features, oxidative stress responses, and clinical prognosis in renal cell carcinoma, underscoring the broad relevance of epigenetic regulation in cancer progression and therapy 22. Enhancers, a key class of cis-regulatory elements, orchestrate gene expression by facilitating promoter activation through transcription factors (TFs) and co-activators, often marked by histone modifications such as H3K27ac and H3K4me1^23^, ^24^. Our previous work demonstrated that enhancer-mediated transcriptional reprogramming contributes to both PTC and anaplastic thyroid carcinoma (ATC) progression, and that targeting super-enhancers through CDK7 inhibition may represent a novel therapeutic approach for ATC 25. These findings support the importance of enhancer-mediated regulatory circuits in thyroid cancer pathogenesis.

In this study, we aimed to systematically investigate the role of BGN in PTC, focusing on its tumor-intrinsic functions and its immunomodulatory effects, particularly in driving macrophage M2 polarization and immune evasion. Furthermore, we explored the enhancer-dependent transcriptional regulation of BGN by NR2F2, elucidating how the NR2F2-BGN axis contributes to tumor progression and immunosuppressive microenvironment formation. Collectively, our work provides mechanistic insights into enhancer-mediated BGN regulation in PTC and highlights the NR2F2-BGN axis as a promising therapeutic target.

## Materials and Methods

### Human tissue and clinical data

The samples and corresponding clinical data used in this study were obtained from patients who underwent surgical treatment at the Department of Thyroid and Neck Tumors, Tianjin Medical University Cancer Institute and Hospital. Informed consent was obtained from all patients prior to surgery. The collection of fresh tissue samples and paraffin-embedded specimens was approved by the Ethics Committee of Tianjin Medical University Cancer Institute and Hospital (Approval No: Ek2021149).

### Database and data analysis

Bulk RNA-seq data and clinical information for PTC tissues were obtained from The Cancer Genome Atlas (TCGA) database. Bulk RNA-seq data from 233 adjacent normal tissues, 18 benign thyroid nodule tissues, and 349 PTC tissues were acquired from the GSE213647 dataset in the Gene Expression Omnibus (GEO) database. Additionally, single-cell RNA sequencing (scRNA-seq) data were analyzed from the GSE250521, GSE193581, and GSE184362 datasets. Spatial RNA sequencing (spRNA-seq) data were sourced from the GSE250521 and HRA003537 datasets 26. Detailed sample information is available in Table S1.

Bulk RNA-seq data were analyzed to assess BGN expression in various tissue types and its correlation with clinical features and prognosis. The scRNA-seq data were processed through conventional dimensionality reduction clustering and annotation with classical marker genes 27-29. The expression of BGN in different cell types was evaluated at the single-cell level, comparing benign and malignant thyroid cells. In the malignant cells of PTC samples, cells with BGN expression of 0 were considered to be BGN- cells, and cells with BGN expression greater than 0 are considered to be BGN+ cells. The “SCP” package was used to calculate the results of the differential analysis between BGN+ malignant cells and BGN- malignant cells and mapped the volcano plot of differential genes. Both P-value < 0.05 and |log_2_FC| > 0.58 were considered significant differential express genes, and they were analyzed by gene set functional analysis. Spatial transcriptomics data were further analyzed to determine the spatial distribution of BGN expression across different tissue types. The spots cell types of sections from the GSE250521 dataset were annotated by RCTD 30 by integrating single-cell and spatial transcriptomics data. While the spatial spots from HRA003537 were annotated following the approach described by Yan *et al* 26.

### BayesPrism deconvolution

BayesPrism deconvolution (v2.0) 31 was used to process bulk RNA-seq data from TCGA and GSE213674 dataset, minimizing the interference of stromal cell signals to isolate the malignant PTC cell-specific expression profiles. In addition, expression profiles of fibroblasts, smooth muscle cells and endothelial cells were extracted from bulk RNA-seq data to analyze the relationship between BGN expression level and clinical features. We utilized the GSE250521 dataset as a reference for the deconvolution process. Default settings were applied throughout the analysis.

### Cell lines and culture

This study used the following cell lines: human thyroid normal cell line Nthy-ori-3, four PTC cell lines (TPC-1, KTC-1, K1, and BCPAP), human leukemia monocyte THP-1 cells, and HEK293T cells. These cell lines were sourced from the American Type Culture Collection (ATCC) and were authenticated using short tandem repeat (STR) profiling to confirm their genetic consistency with the original cells. And the murine thyroid cancer cells were obtained as previously described 32.

The culture medium for BCPAP, KTC-1, K1, and THP-1 cells was consisted of 90% RPMI-1640 medium, 10% fetal bovine serum (FBS), and 1% penicillin-streptomycin. Nthy-ori 3-1, TPC-1, murine thyroid cancer cells, and HEK293T cells were cultured in 90% DMEM medium, 10% FBS, and 1% penicillin-streptomycin. All cells were maintained in a 37 °C, 5% CO₂ incubator, and media changes, passaging, and freezing were performed as necessary.

### Cell transfection

THP-1 cells were seeded in 6-well plates at a density of 2 × 10^5^ cells/well with 2 mL of culture medium containing 10% FBS. The cells were cultured at 37 °C, 5% CO₂ until they reached 60%-70% confluence. Lentivirus was transfected into the target cells, followed by selection in culture medium containing antibiotics to establish stable BGN knockdown or overexpression cell lines. After one week of culture, RNA and protein were extracted from the cells to verify the successful construction of the cell lines. The sequences of the target shRNA and sgRNA plasmids are provided in **Table S2**.

For transient transfection, specific siRNAs targeting the gene of interest (sequences provided in **Table S2**) and negative control siRNA were used. Transfection complexes were prepared in a sterile 1.5 mL EP tube by mixing 5 μL siRNA (20 μM) with 245 μL serum-free medium. Next, 5 μL of Lipofectamine 2000 and 240 μL of serum-free medium were mixed and incubated at room temperature for 5 minutes. The transfection complex was added to each well, and cells were cultured for 48-72 h post-transfection.

### Quantitative real-time PCR

Total RNA from tissues or cells was extracted using TRIzol reagent (SparkZol) and quantified with a spectrophotometer to assess RNA concentration and purity. Reverse transcription was performed using a reverse transcription kit (Vazyme) to synthesize cDNA. Specific primers and SYBR Premix Ex Taq II reagent were used for fluorescence quantitative PCR. The primer sequences are listed in **Table S3**.

### Western blotting analysis

Protein extraction was performed on tissues or adherent cells using RIPA lysis buffer (Beyotime) supplemented with protease inhibitors (Beyotime). Proteins were separated by SDS-PAGE and transferred to PVDF membranes. Membranes were blocked with 5% non-fat milk and incubated with primary antibodies diluted in antibody dilution buffer overnight at 4 °C. The following primary antibodies were used: BGN (1:1000, Cat:16409-1-AP, Proteintech, USA), β-actin (1:1000, Cat#UM4001, UTIBODY, China), GAPDH (1:1000, Cat#UM4002, UTIBODY, China), AKT (1:1000, Cat#4060, CST, USA), p-AKT (1:1000, Cat#ab8805, Abcam, USA), Flag (1:1000, Cat#14793, CST, USA). After incubation with the primary antibody, membranes were incubated with secondary antibodies, and protein bands were detected using an enhanced chemiluminescence system.

### Tissue immunohistochemistry

Tissue sections were dewaxed and rehydrated using a 65 °C oven overnight. Antigen retrieval was performed with EDTA antigen retrieval solution. Sections were blocked with 3% H₂O₂ for 30 min. After washing with PBS, the sections were incubated overnight at 4 °C with primary antibodies: BGN (1:200-1:500, Cat:16409-1-AP, Proteintech, USA), NR2F2 (1:100, Cat#A24647, Abclonal, China), Ki67 (1:500, Cat:9449, CST, USA), and p-AKT (1:200, Cat#ab8805, Abcam, USA). The next day, sections were incubated with secondary antibodies for 1 hour at room temperature. After DAB staining, sections were counterstained with hematoxylin, dehydrated, cleared, and mounted. Three random fields per slide were analyzed for immunohistochemical scoring using an optical microscope.

### Recombinant biglycan (rBGN)

The recombinant Biglycan (rBGN) protein used in this study was purchased from Proteintech (Cat No. Ag8012). The sequence of rBGN is as follows:

MWPLWRLVSLLALSQALPFEQRGFWDFTLDDGPFMMNDEEASGADTSGVLDPDSVTPTYSAMCPFGCHCHLRVVQCSDLGLKSVPKEISPDTTLLDLQNNDISELRKDDFKGLQHLYALVLVNNKISKIHEKAFSPLRKLQKLYISKNHLVEIPPNLPSSLVELRIHDNRIRKVPKGVFSGLRNMNCIEMGGNPLENSGFEPGAFDGLKLNYLRISEAKLTGIPKDLPETLNELHLDHNKIQAIELEDLLRYSKLYRLGLGHNQIRMIENGSLSFLPTLRELHLDNNKLARVPSGLPDLKLLQVVYLHSNNITKVGVNDFCPMGFGVKRAYYNGISLFNNPVPYWEVQPATFRCVTDRLAIQFGNYKK.

### Cell viability CCK-8 assay

PTC stable cell lines were seeded in 96-well plates at a density of 1.5 × 10^3^ cells/well in 100 μL medium with 10% FBS. After 12 h of culture at 37 °C, 5% CO₂, the cells were treated with 200 μL medium containing 10% FBS and rBGN protein (0.2 μg/mL). Absorbance was measured at 24, 48, 72, and 96 h after adding 10 μL CCK-8 reagent. Absorbance (OD) was measured at 450 nm, and cell proliferation was calculated.

### Colony formation assay

PTC cells were seeded in 6-well plates at a density of 800 cells/well with 2 mL of medium containing 10% FBS. After 10-14 days, cells were fixed with 4% formaldehyde, stained with 0.1% crystal violet for 30 min, and washed with distilled water. Colonies were photographed, and the number of colonies was counted using ImageJ software to calculate the colony formation rate.

### Cell cycle assay

PTC cells were seeded in 6-well plates at a density of 2 × 10^5^ cells/well with 2 mL of medium containing 10% FBS. For the rBGN group, recombinant BGN protein (0.2 μg/mL) was added. After the cells reached 80%-90% confluence, cells were fixed with 70% ethanol overnight at 4 °C. After staining with PI and RNase A, cell cycle distribution was analyzed using flow cytometry.

### Conditional medium from PTC cells

Stable PTC cells were seeded at a density of 2 × 10^5^ cells/well in 6-well plates. After reaching appropriate confluence, the cells were washed with serum-free RPMI-1640 medium and then cultured in fresh serum-free medium for 24 h. The conditioned medium (CM) was collected, mixed with an equal volume of medium containing 10% FBS, and stored at -80 °C.

### THP-1 derived macrophages transwell migration assay

THP-1 cells were seeded at 2 × 10^5^ cells/well in 6-well plates, differentiated into macrophages by treatment with 20 ng/mL PMA for 48 h. The differentiated macrophages were incubated with an equal volume of CM for 48 h, and then their migration ability was assessed using a transwell assay.

### Flow cytometry

After 48 h of co-incubation with the conditioned medium (CM), the induced macrophages were digested with trypsin. The digestion was terminated by adding medium containing 10% FBS, and the cell suspension was transferred to a centrifuge tube for centrifugation. The cells were then resuspended in 100 μL of PBS and stained with 5 μL of Zombie Nir-APC/Cy7 antibody (Cat#423106, Biolegend, USA) for 30 minutes in the dark. Next, 5 μL of CD86-FITC (Cat#374204, Biolegend, USA) and 5 μL of CD206-PE (Cat#321106, Biolegend, USA) were added, and the cells were incubated for 30 minutes in the dark. After fixation with Fixation Buffer (Cat#88-8824-00, Thermo Fisher, USA), the cells were permeabilized with permeabilization solution. Finally, 5 μL of CD68-PE/Cy7 antibody (Cat#321106, Biolegend, USA) was added, and the cells were incubated for 30 minutes at room temperature in the dark. The cells were then fixed with 1% paraformaldehyde and analyzed using flow cytometry.

### RNA-seq analysis

RNA was extracted from PTC cells with stable BGN knockdown and control cells using TRIzol reagent. Sequencing was performed by Beijing Tiangen Biotech, and quality control was carried out with FastQC and Trimmomatic. Clean reads were mapped to the hg38 reference genome using STAR, and gene expression levels were quantified using featureCounts. Differentially expressed genes (|log2FoldChange| > 1.5, *p* < 0.05) were identified by DESeq2 and analyzed through GO, KEGG, and GSEA. Pathway activity was assessed using decoupleR.

### ChIP and ChIP-qPCR

After culturing the cells to 80-90% confluence, they were cross-linked with 1% formaldehyde and the reaction was terminated. The cells were then collected, centrifuged, and the pellet was harvested. Next, the nuclei were isolated using a buffer solution, and chromatin was digested with Micrococcal Nuclease to generate appropriately sized fragments. The cells were subsequently sonicated. Afterward, centrifugation and DNA purification were performed to assess the digestion and concentration of the chromatin.

During the immunoprecipitation (IP) step, each sample was incubated overnight with 2-3 μg of H3K27ac antibody (Cat#ab4729, Abcam, USA), BRD4 antibody (Cat#13440, CST, USA), Flag antibody (Cat#14793, CST, USA), and CTCF antibody (Cat#3418S, CST, USA) followed by immunoprecipitation with magnetic beads. The samples were washed with a series of buffer solutions to remove non-specific binders. After chromatin elution, the cross-links were reversed under high-temperature conditions, and DNA was extracted using a DNA purification kit. The purified DNA was then used for subsequent experiments, such as qPCR analysis.

### ChIP-seq data processing

ChIP-seq reads were aligned to the human reference genome (hg38) using Bowtie2 (v2.2.5). Unmapped and non-uniquely mapped reads were removed with SAMtools (v1.20), and PCR duplicates were filtered out using GATK (v4.6.0). The bamCoverage tool (v3.5.5) was employed to convert BAM files into bigWig format, representing read coverage across genomic regions. Peak calling for the CTCF ChIP-seq dataset, relative to the Input control, was performed using MACS2 (v2.2.9.1) with a p-value threshold of 1 × 10⁻⁵. EnrichedHeatmap (v1.32.0) was applied to visualize read enrichment intensity within ±10.0 kb of transcription start sites (TSSs). Genomic annotation of CTCF peaks was conducted using the “ChIPSeeker” package (v1.38.0).

### Chromosome conformation capture (3C) assay

The cells were prepared by washing with PBS, followed by trypsin digestion and centrifugation to collect the cell pellet. The cells were cross-linked with formaldehyde, and the reaction was allowed to shake for 8 minutes, after which glycine was added to terminate the cross-linking. The cells were washed and stored for future use. Next, 3C buffer and PMSF were added, and the cells were incubated on ice for 30 minutes, followed by centrifugation. The cell pellet was washed with NEB buffer, and then subjected to digestion and ligation overnight.

The samples were treated with Proteinase K to extract and purify the nucleic acids. DNA quality was assessed by agarose gel electrophoresis. The ligation products were further processed by incubation, Proteinase K treatment, nucleic acid recovery, cross-link reversal, and phenol-chloroform extraction to obtain the 3C library. Detailed protocols for these steps can be found in the protocols by Dekker and Zhang *et al* 33, 34. The constructed 3C library was used for subsequent PCR experiments. The specific 3C primers designed for the BGN enhancer and promoter regions are provided in **Table S4**.

### CRISPR/Cas9-mediated interference of BGN enhancer

The sgRNA sequences were designed and modified using Zlab design software, with BsmBI-V2 enzyme recognition sites added at both ends of the sequences. The sequence information is provided in **Table S5**. Next, an enzymatic digestion reaction was prepared to digest the Lenti samV2 KRAB template plasmid. Following digestion, the sgRNA oligos were annealed using a gradual temperature reduction method through a thermal cycler to complete the annealing reaction. The digestion products were excised from the gel under UV light and purified using a gel extraction kit. The purified products were then ligated under the following conditions: 16°C incubation for 8 hours. The ligation products were used to transform competent cells, and the transformed cells were cultured for plasmid amplification. The plasmids were subsequently sent for sequencing, and sequence verification was performed using SnapGene software. Finally, the successfully verified plasmids were used for subsequent CRISPR interference (CRISPRi) steady-state construction.

### Luciferase reporter assay

PTC cells were seeded into 24-well plates and cultured until they reached 60% - 70% confluence, at which point the transfection complex was prepared. The transfection complex was composed of 0.5 μg reporter plasmid, 0.05 μg Renilla plasmid, 0.5 μg expression plasmid, and Lipofectamine 2000. The components were mixed and incubated at room temperature for 20 minutes. The transfection complex was then added to the cells, and the cells were cultured for 48 hours. After transfection, the medium was discarded, and the cells were washed with PBS. Cells were then lysed using 1× Passive Lysis Buffer, and the supernatant was collected by centrifugation. Firefly luciferase activity was measured using the Luciferase Assay Kit, followed by Renilla luciferase activity detection using the Stop & Glo assay kit. The relative activity of the reporter gene was calculated by determining the ratio of Firefly luciferase activity to Renilla luciferase activity (Firefly/Renilla).

### Animal experiments

All animal care and experimental protocols were approved by the Ethics Committee of Tianjin Medical University Cancer Institute and Hospital, in accordance with the standards of the U.S. National Institutes of Health (Approval No. AE-2022032).

Macrophage depletion: One day prior to subcutaneous tumor inoculation, each mouse received an intraperitoneal injection of 50 µL clodronate liposomes. Subsequent injections of 20 µL clodronate liposomes were administered intraperitoneally every other day until the end of the experiment. The control group received an equal volume of PBS liposomes.

Subcutaneous tumor implantation in C57BL/6J Mice: After one week of acclimatization, C57BL/6J mice were randomly assigned to groups and subcutaneously implanted with approximately 4 × 10^6^ murine thyroid cancer cells transfected with Nr2f2 overexpression or Bgn knockdown. Tumor volume was measured 2-3 times every 3 days using calipers to measure the longest (L) and shortest (W) diameters, and calculated using the formula V = (L × W²)/2.

CIA1 drug inhibition: Once murine thyroid tumors were established, mice were intraperitoneally injected with 2.5 mg/kg CIA1 drug every other day. Tumor volume changes were recorded, and tumor growth curves were generated. After 4 weeks, mice were euthanized, and tumors, along with heart, liver, and kidneys, were harvested for hematoxylin and eosin (H&E) staining. A portion of the tumor tissue was fixed in 4% paraformaldehyde for histological analysis and used for flow cytometry.

Co-injection model in nude mice: BALB/c nude mice were randomly divided into groups. Each mouse was subcutaneously injected with approximately 1 × 10^7^ K1 stable cells and 2 × 10^6^ THP-1 derived macrophages 35, 36.

Flow cytometry analysis of subcutaneous tumor tissues from C57BL/6J mice and the co-injection model of nude mice was performed. Tumor tissues were enzymatically digested with 1 mg/mL type IV collagenase and 50 μg/mL DNase I to dissociate the tissue and release cells. After digestion, single-cell suspensions were passed through a 70 μM cell strainer to remove non-cellular debris, ensuring sample purity. A red blood cell lysis step was then performed to further eliminate residual erythrocytes. To distinguish live and dead cells, Zombie Nir dye was used to stain the cells, and flow cytometry was applied to select viable cells for further analysis. The panel of fluorescently-conjugated monoclonal antibodies (CD45-PerCP/Cy5, CD11b-PE/Cy7, F4/80-FITC, CD86-PE, and CD206-APC) were used to identify infiltrated macrophages in murine subcutaneous tumor from C57BL/6J. The number of infiltrating macrophages (CD11b+F4/80+) and the proportions of M1 (CD86+CD206-) and M2 (CD86-CD206+) macrophages were analyzed to evaluate their polarization status. We employed a panel of fluorophore-conjugated monoclonal antibodies, including CD45-PerCP/Cy5, CD68-PE/Cy7, CD86-FITC, and CD206-PE to identify macrophage in the tumor tissue co-injected with K1 cells and THP-1 cells, and the proportion of M1-type (CD86⁺) and M2-type (CD206⁺) macrophages was determined via flow cytometry to assess their polarization state.

### Statistical analysis

Data were analyzed using GraphPad Prism (v8.3.0), R (v4.3.1), and ImageJ (v1.8.0). Statistical comparisons were made using t-tests, ANOVA, or non-parametric tests as appropriate. Correlations were evaluated with Spearman's rank correlation coefficient. Statistical significance was set at *p* < 0.05.

## Results

### High BGN expression in PTC tissues is significantly associated with poor prognosis

**Figure 1:**
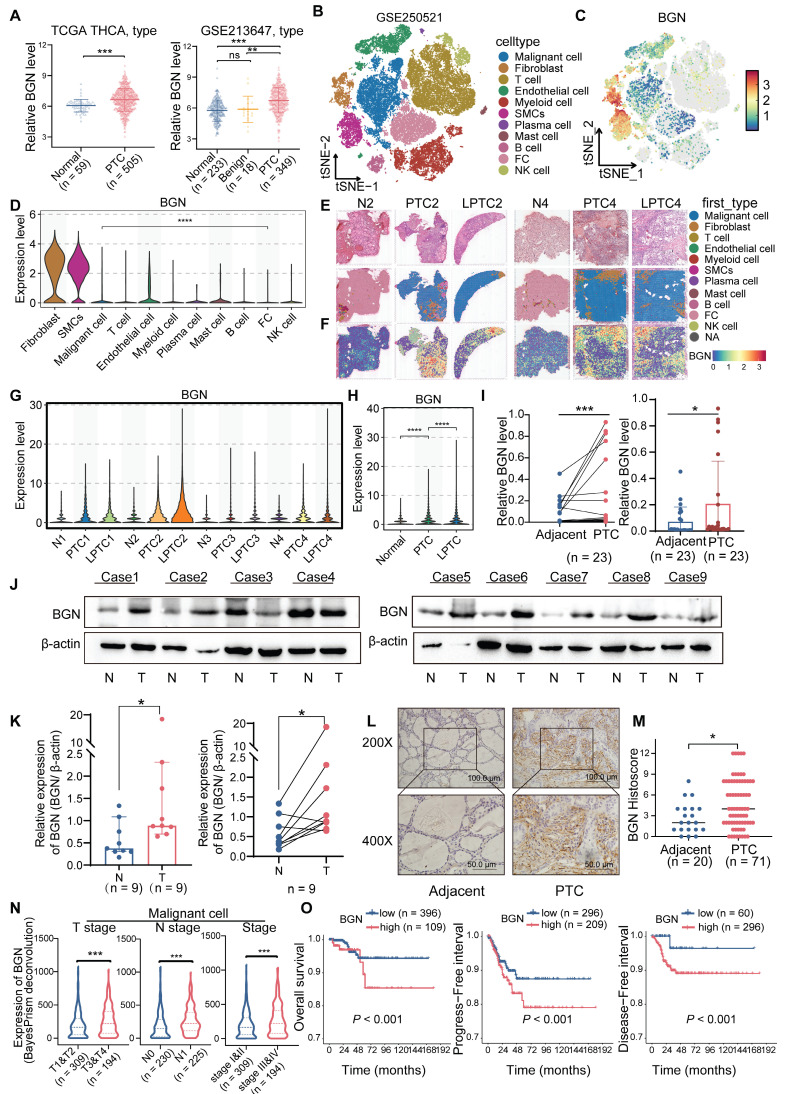
**BGN is highly expressed in PTC and correlates with advanced clinical outcomes. (A)** the mRNA expression levels of BGN in the TCGA and GEO databases (left: THCA dataset from TCGA; right: GSE213647 dataset from GEO).** (B)** t-SNE plot of cell clusters from spatial transcriptomics dataset GSE250521, annotated by cell types. (**C**) Distribution of BGN expression across different cell clusters in the t-SNE scatter plot. **(D)** Violin plot showing BGN expression levels across major cell types in scRNA-seq data. **(E-F)** Spatial transcriptomic maps showing histology (E) and BGN expression (F) in PTC, LPTC, and adjacent normal tissues. **(G)** Violin plot showing BGN expression levels in follicular cells and malignant tumor cells spots among each sample by spatial transcriptomic analysis. **(H)** Violin plot showing BGN expression levels in follicular cells and malignant tumor cells spots among adjacent normal, PTC, and LPTC sections by spatial transcriptomic analysis. **(I)** qRT-PCR analysis of BGN mRNA levels in paired and grouped PTC tissues and adjacent normal tissues. **(J-K)** Western blot analysis of BGN protein levels in PTC tumor tissues and adjacent normal tissues. (**L**) Representative immunohistochemical (IHC) staining images of BGN protein expression in adjacent normal and PTC tissues (scale bar: 50 μM at 200×, 100 μM at 400× magnification).** (M)** Comparison of IHC scores for BGN protein expression between adjacent normal and PTC tissues. **(N)** Violin plots showing differences in relative BGN expression levels in malignant cells, as determined by BayesPrism deconvolution analysis, among different clinicopathological subgroups. **(O)** Kaplan-Meier survival analysis demonstrates that high malignant cell-derived BGN expression, classified by the optimal cutoff from BayesPrism deconvolution analysis, is associated with poorer overall survival (OS), progression-free survival (PFS), and disease-free survival (DFS) in PTC patients. (*p < 0.05, **p < 0.01, ***p < 0.001).

To investigate the expression characteristics of BGN in PTC tissues, we integrated multi-omics datasets. The results demonstrated that the mRNA levels of BGN were significantly higher in PTC tissues compared to adjacent normal tissues, whereas no significant difference was observed between adjacent normal tissues and benign thyroid nodules (Figure 1A). BGN has been widely reported as a biomarker for CAFs 37. To better elucidate the cell-type-specific expression pattern of BGN, we performed an in-depth analysis of three independent single-cell RNA sequencing datasets (Figure 1B and Figure S1A-F) 27-29. Among the annotated cell types, BGN expression was notably higher in stromal cells, such as fibroblasts, endothelial cells, and smooth muscle cells (SMCs) (Figure 1C and Figure S1G-L). Although BGN expression in malignant tumor cells was lower than that in stromal populations, its expression in tumor cells remained notably high and should not be overlooked, particularly when compared to normal thyroid follicular cells, where BGN expression was significantly lower (Figure 1D and Figure S1I, L).

To assess the spatial distribution of BGN in PTC tissues, we employed spatial transcriptomics data from dataset GSE250521, where spot annotations were inferred using matched scRNA-seq references. Spatial mapping revealed that BGN expression was concentrated in regions enriched with stromal and malignant PTC cells (Figure 1E-F and Figure S1M). Comparative expression analysis across annotated spot types demonstrated that BGN expression was significantly lower in para-tumoral normal tissues than in both PTC and locally advanced PTC (LPTC) samples (Figure 1G-H). Further validation was performed using an additional published spatial transcriptomics dataset for consistency (Figure S1N-O). Across all five analyzed tissue sections, BGN expression was significantly elevated in malignant tumor spots relative to adjacent normal follicular cell spots (Figure S1P-Q).

To validate these findings in clinical specimens, we measured BGN mRNA levels in tumor and matched adjacent normal tissues from 23 PTC patients. Both paired and independent analyses confirmed a significant upregulation of BGN in tumor tissues (Figure 1I). Consistently, Western blot analysis of protein lysates from nine PTC patients showed that seven patients exhibited markedly higher BGN protein levels in tumors compared to adjacent normal tissues (Figure 1J-K). Additionally, immunohistochemical (IHC) staining of formalin-fixed paraffin-embedded (FFPE) samples from 71 PTC patients (including 20 paired samples) revealed significantly stronger BGN staining in tumor tissues (Figure 1L-M).

Our results demonstrated that BGN expression in tumor cells and fibroblasts was positively correlated with later T stage and TNM stage, whereas BGN expression in endothelial cells and smooth muscle cells was inversely correlated with aggressiveness. In terms of N stage, BGN secreted by tumor cells was highly expressed in the lymph node metastasis group, but BGN secreted by fibroblasts was not related to N stage. Importantly, patients with high BGN expression in tumor cells or fibroblasts exhibited significantly poorer prognosis, while high BGN expression in endothelial or smooth muscle cells correlated with better survival (Figure 1N-O and Figure S1R-X). This suggests that BGN secreted by tumor cells may be involved in tumor progression while the BGN secreted by endothelial cells and smooth muscle cells has a different function in PTC.

### BGN promotes PTC progression by activating the AKT signaling pathway

**Figure 2:**
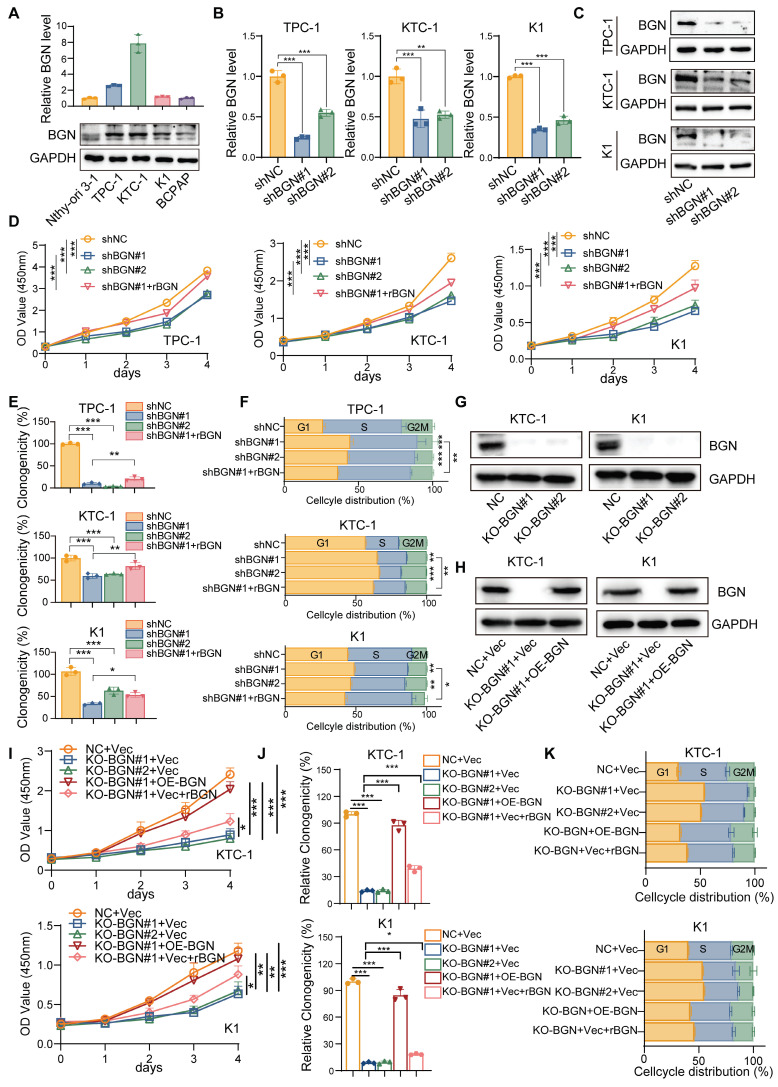
** The BGN level of PTC cell altered the proliferation and cell cycle distribution. (A)** qRT-PCR and Western blot analyses of BGN mRNA and protein expression levels in multiple PTC cell lines. **(B)** qRT-PCR analysis of BGN mRNA levels in TPC-1, KTC-1, and K1 cells following BGN knockdown. **(C)** Western blot analysis of BGN protein levels in TPC-1, KTC-1, and K1 cells after BGN knockdown. **(D)** CCK-8 assay assessing relative cell viability in TPC-1, KTC-1, and K1 cells upon BGN knockdown or rBGN supplement. **(E)** Colony formation assays evaluating the proliferative capacity of TPC-1, KTC-1, and K1 cells following BGN knockdown or rBGN supplement. **(F)** Flow cytometry analysis of cell cycle distribution in TPC-1, KTC-1, and K1 cells upon BGN knockdown or rBGN supplement. **(G-H)** Western blot analysis of BGN expression in KTC-1 and K1 cells with CRISPR-Cas9-mediated BGN knockout (G) or BGN overexpression in the BGN knockout stable cell lines. (**I**) CCK-8 assay assessing relative cell viability in KTC-1 and K1 cells upon BGN knockout, BGN overexpression or rBGN supplement. (**J**) Colony formation assays evaluating the proliferative capacity of KTC-1 and K1 cells following BGN knockout, BGN overexpression or rBGN supplement. (**K**) Flow cytometry analysis of cell cycle distribution in KTC-1 and K1 cells following BGN knockout, BGN overexpression or rBGN supplement. (*p < 0.05, **p < 0.01, ***p < 0.001).

To further investigate the biological function of BGN in PTC, we performed both shRNA-mediated BGN knockdown and CRISPR-Cas9-mediated BGN knockout in PTC cell lines with relatively higher endogenous BGN levels (Figure 2A). Stable BGN-knockdown thyroid cancer cell lines were generated using lentiviral infection (Figure 2B-C). CCK-8 and colony formation assays demonstrated that BGN silencing significantly inhibited PTC cell proliferation and colony formation. Notably, supplementation with human recombinant BGN protein (rBGN) partially restored both proliferative activity and colony formation capacity in BGN-knockdown cells (Figure 2D-E and Figure S2A). Cell cycle analysis via flow cytometry revealed that BGN knockdown induced G1 phase arrest in PTC cells, while rBGN supplementation partially reversed this effect, indicating a critical role for BGN in cell cycle regulation (Figure 2F and Figure S2B). To obtain more reliable rescue experiments, we employed CRISPR-Cas9-mediated BGN knockout and subsequently re-expressed BGN in the knockout cell lines to elucidate the specific role of BGN (Figure 2G-H). Additionally, rBGN was supplemented into the BGN knockout cells. The results demonstrated that both BGN overexpression and rBGN supplementation effectively rescued the decrease in cell viability (Figure 2I), restored proliferative capacity (Figure 2J and Figure S2C), and alleviated the G1/S phase arrest (Figure 2K and Figure S2D) induced by BGN knockout. These findings strongly suggest that BGN levels play a crucial role in regulating cell proliferation and cell cycle progression in PTC cells.

**Figure 3:**
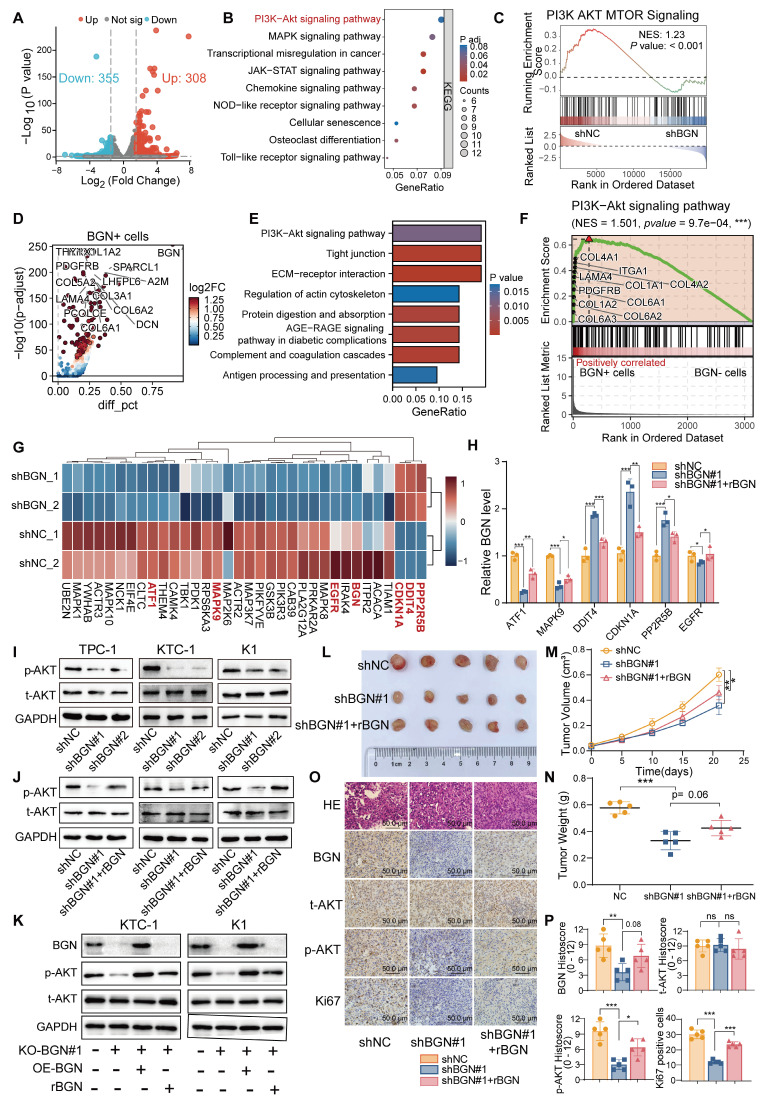
**BGN promotes PI3K/AKT pathway activation and facilitates PTC tumorigenesis. (A)** Volcano plot illustrating differentially expressed genes following BGN knockdown. **(B)** KEGG pathway analysis showing significantly enriched signaling pathways among downregulated genes in the shBGN group.** (C)** GSEA analysis demonstrating that the PI3K-AKT-mTOR signaling pathway is significantly enriched in the shNC group compared to the shBGN group. **(D)** Volcano plot of differentially expressed genes between BGN⁺ and BGN⁻ cells in malignant PTC cells of PTC samples from single-cell RNA-seq data, annotated the top 15 upregulated genes in BGN⁺ cells. **(E)** KEGG analysis of genes upregulated in BGN⁺ cells. **(F)** GSEA analysis showed the PI3K-AKT signaling pathway enriched in BGN⁺ cells.** (G)** Heatmap displaying the expression levels of key genes in the AKT signaling pathway. **(H)** qRT-PCR validation of key AKT signaling pathway genes in the shBGN#1 and shBGN#1+rBGN groups. **(I-J)** Western blot analysis of p-AKT levels upon BGN knockdown (I) and exogenous rBGN supplementation (J). **(K)** Western blot analysis of p-AKT levels upon BGN knockout, BGN overexpression or rBGN supplementation. **(L)** Schematic representation of the xenograft tumor model and representative images of tumor tissues. **(M)** Tumor growth curves of control (shNC), BGN knockdown (shBGN#1), and rBGN-treated (shBGN#1+rBGN) groups in a subcutaneous xenograft model. **(N)** Comparison of tumor sizes among different treatment groups. **(O)** Representative IHC staining images of BGN, Ki67, total AKT and p-AKT in xenograft tumor tissues from each group.** (P)** Quantification of BGN, Ki67, total AKT and p-AKT expression levels in xenograft tumor tissues across different groups. (*p < 0.05, **p < 0.01, ***p < 0.001).

To further elucidate the molecular mechanisms underlying BGN function, RNA sequencing (RNA-seq) was performed on KTC-1 cells with BGN knockdown to identify differentially expressed genes (DEGs). A total of 308 genes were significantly upregulated, while 355 genes were downregulated in shBGN cells compared to controls (Figure 3A). Gene Ontology (GO) enrichment analysis revealed that upregulated genes in the shBGN group were primarily associated with ncRNA processing, ribonucleoprotein complex biogenesis, ribosome biogenesis, and DNA replication (Figure S2A). In contrast, downregulated genes were significantly enriched in biological processes related to leukocyte and macrophage chemotaxis and migration (Figure S2B). KEGG pathway analysis further revealed that downregulated DEGs were significantly enriched in critical signaling pathways, including the PI3K-Akt, MAPK, NOD-like receptor, and Toll-like receptor pathways (Figure 3B). Additionally, Gene Set Enrichment Analysis (GSEA) indicated that the PI3K-AKT-MTOR pathway was significantly enriched in the shNC control group compared to the shBGN group (Figure 3C). To further validate these findings, we analyzed pathway activity using the “decoupleR” software. The results demonstrated that PI3K/AKT pathway activity was markedly reduced in the shBGN group compared with the shNC group (Figure S3C).

To assess the relevance of these transcriptomic changes in a clinical context, we analyzed scRNA-seq data from malignant PTC cells. Volcano plot analysis comparing BGN⁺ and BGN⁻ PTC cells identified the upregulated DEGs in BGN⁺ cells (Figure 3D). And the KEGG enrichment analysis revealed significant activation of PI3K-AKT signaling in BGN⁺ malignant PTC cells (Figure 3E). The GSEA analysis showed marked enrichment of PI3K-AKT signaling in BGN⁺ malignant PTC cells (Figure 3F). The PI3K/AKT signaling pathway plays a pivotal role in PTC tumorigenesis 38. Further analysis revealed that key inhibitory genes in the AKT signaling pathway (DDIT4, PPP2R5B) and the downstream cell cycle regulator CDKN1A (p21) were upregulated upon BGN knockdown, whereas activator genes (ATF1, MAPK9) were downregulated (Figure 3G). These findings suggest that BGN may contribute to PTC tumorigenesis by modulating the PI3K/AKT pathway. To validate these results, we first confirmed the expression levels of these key genes via qRT-PCR (Figure 3H). Subsequently, Western blot analysis revealed a significant reduction in phosphorylated AKT levels following BGN knockdown, while rBGN supplementation reversed this effect (Figure 3I-J). Furthermore, AKT activation was enhanced in cells with BGN overexpression or rBGN supplementation following the knockout of BGN (Figure 3K).

To assess the biological function of BGN *in vivo*, we established a subcutaneous xenograft tumor model in nude mice using K1 cells (Figure 3L). Tumor growth curves indicated that the growth rate of BGN-silenced tumors was significantly reduced compared to the control group (Figure 3M). At the experimental endpoint, tumor dissection and weighing revealed that the tumor weight in the BGN-knockdown group was significantly lower than in the control group (Figure 3N). Notably, supplementation with rBGN (0.2 μg/mL) in BGN-knockdown K1 cells prior to subcutaneous injection partially restored tumor growth in nude mice (shBGN#1 + rBGN group) (Figure 3L-N). Immunohistochemical staining of tumor tissues demonstrated a positive correlation between p-AKT expression and BGN levels, with a similar trend observed in Ki67-positive cell rates (Figure 3O-P). These findings collectively suggest that BGN may promote PTC tumor growth by activating the AKT signaling pathway, thereby playing a crucial role in PTC progression.

### BGN shapes the immunosuppressive tumor microenvironment by promoting M2 macrophage polarization via TLR4 signaling in PTC

**Figure 4:**
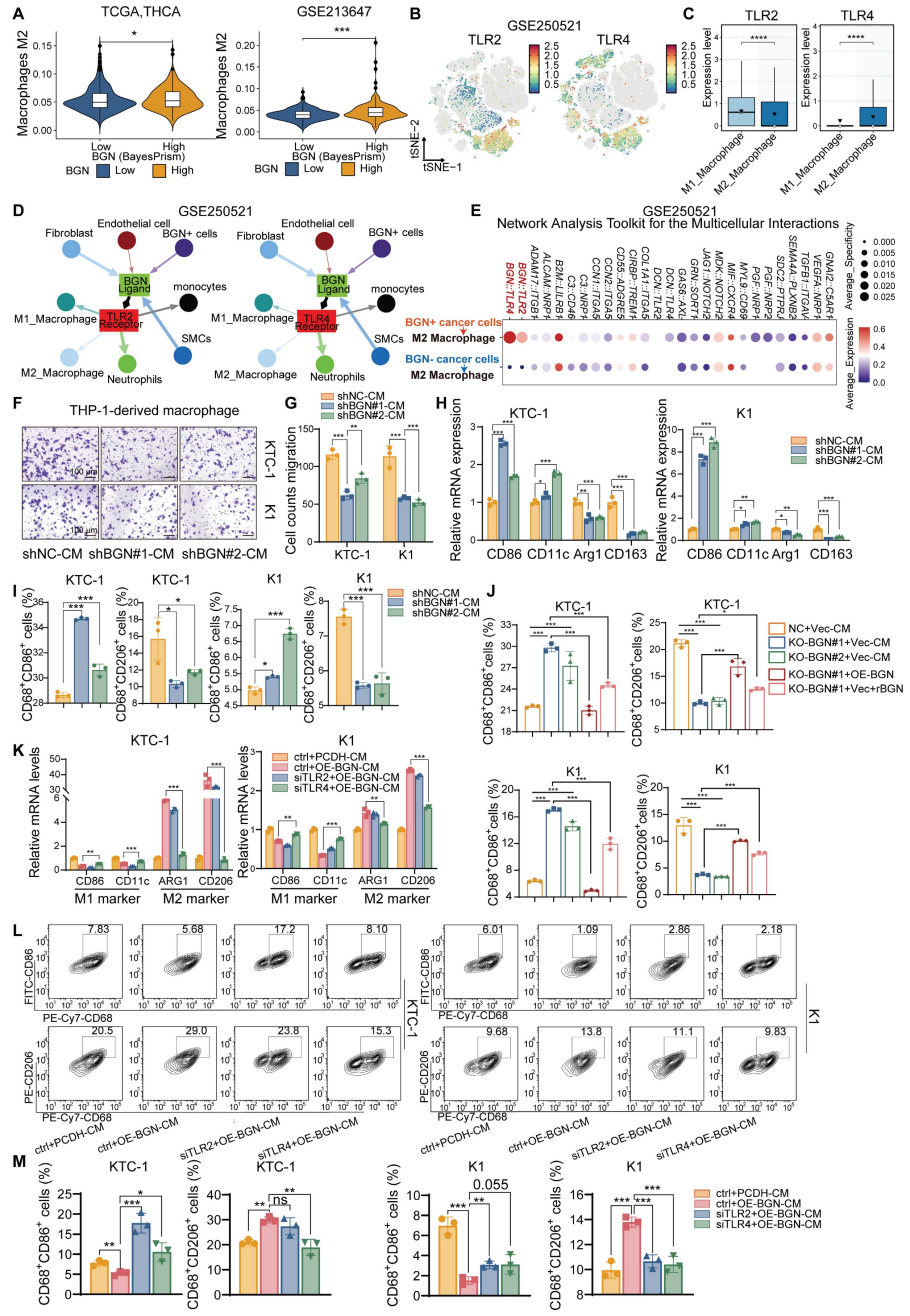
** PTC promotes M2 polarization of macrophages through BGN/TLR4 signaling. (A)** Correlation between BGN expression in malignant cells, as determined by BayesPrism deconvolution analysis, and M2 macrophage infiltration levels, quantified using the quanTIseq method. **(B)** t-SNE scatter plot showing the distribution of TLR2 (left) and TLR4 (right) in the GSE250521 scRNA-seq dataset. **(C)** Box plot showing the expression levels of TLR2 and TLR4 in M1 and M2 macrophages from the GSE250521 datasets. **(D)** The interaction network between BGN ligands and TLR2/TLR4 receptors in a cell subset in the GSE250521 dataset (the length of the arrow represents the interaction edge of the receptor and ligand pair, and the thickness of the arrow represents the expression intensity of the receptor and ligand pair). **(E)** Bubble plot displaying the ligand-receptor interactions between BGN+ tumor cells and M2 macrophages in the GSE250521 dataset by NATMI. **(F-G)** Conditioned medium (CM) from BGN-knockdown KTC-1 or K1 cells inhibits the migration ability of THP-1-derived macrophages (scale bar: 100 μM). **(H)** qRT-PCR analysis of M1 macrophage markers (CD86, CD11c) and M2 macrophage markers (Arg1, CD163) in macrophages co-cultured with CM from control and BGN-knockdown groups.** (I)** Flow cytometry analysis of M1 and M2 macrophage proportions in macrophages co-cultured with CM from control and BGN-knockdown groups.** (J)** Flow cytometry analysis of M1 and M2 macrophage populations after incubation with CM from the BGN knockout group compared to the control group, and with CM from the BGN overexpression and rBGN supplementation groups compared to the BGN knockout group.** (K)** qRT-PCR analysis of M1 (CD86, CD11c) and M2 (Arg1, CD163) macrophage markers in macrophages co-cultured with CM from control and BGN-overexpression groups after TLR2 or TLR4 knockdown. **(L)** Representative flow cytometry results of M1 (CD68+CD86+) and M2 (CD68+CD206+) macrophages after co-culture with CM from control and BGN-overexpression groups following TLR2 or TLR4 knockdown. **(M)** Flow cytometry analysis quantifying the proportions of M1 and M2 macrophages in macrophages co-cultured with CM from control and BGN-overexpression groups after TLR2 or TLR4 knockdown. (*p < 0.05, **p < 0.01, ***p < 0.001).

As an ECM component, BGN can act as a damage-associated molecular pattern (DAMPs) and was the key regulator of macrophage activation 39, 40. It has also been reported to reshape the TME by modulating immune cell infiltration and function, thereby contributing to immune evasion 41. To further investigate the potential association between BGN and the TME, we performed “ESTIMATE” 42 to evaluate the immune score and tumor purity using bulk RNA-seq data from two PTC cohorts: TCGA-THCA and GSE213647. In both datasets, patients with advanced clinical features (extrathyroidal extension, T3/4 stage, lymph node metastasis, or TNM stage III/IV) exhibited significantly higher immune scores and lower tumor purity (Figure S4A-B). Notably, PTC patients with high BGN expression secreted by malignant PTC cells demonstrated elevated immune scores and markedly reduced tumor purity compared to those with low BGN expression (Figure S4C), suggesting a possible link between BGN and increased immune cell infiltration. “BayesPrism” was applied to bulk RNA-seq data to extract tumor cell-specific expression profiles, enabling us to assess the relationship between BGN expression of malignant cells and immunosuppressive signatures. We analyzed its association with multiple immunosuppressive genes. In both datasets, BGN expression showed the stronger positive correlation with CD276 (R > 0.6, *P* < 0.001) (Figure S4D). Collectively, these results support the hypothesis that BGN may play a pivotal role in shaping an immunosuppressive TME in PTC. Interestingly, CD276, derived from both tumor and macrophage sources, has been shown to play a key role in tumor immune evasion 43 Furthermore, PTC patients with high BGN expression demonstrated a lower response rate to immune checkpoint blockade (ICB) therapy than those with low BGN expression, suggesting that elevated BGN levels may impair the efficacy of ICB in thyroid cancer (Figure S4E). Consistently, M2 macrophages infiltration score quantified by the “quanTIseq” method 44 was significantly higher in the BGN-high group compared to the BGN-low group, reinforcing the potential role of BGN in promoting an immunosuppressive macrophage-dominant TME (Figure 4A).

To gain insights into how BGN modulated the immune microenvironment, we analyzed scRNA-seq data. Compared to adjacent normal thyroid tissues, PTC tissues showed a marked increase in myeloid cell proportions and a significant reduction in T cell proportions (Figure S4F-G). Given prior reports that BGN promoted myeloid-derived suppressor cells (MDSCs) infiltration in prostate cancer and is associated with M2 macrophages and Tregs infiltration in colorectal cancer 41, 45. Therefore, BGN may also regulate specific myeloid cell subsets in the PTC TME. Tumor-associated macrophages (TAMs), particularly M2-polarized macrophages, are key players in promoting immune evasion, angiogenesis, and metastasis 46.

BGN has been extensively studied as a natural ligand of TLR2/TLR4 receptors and an important inflammation-related factor in inflammation-related diseases 47-49. We classified myeloid cells into M1 macrophages, M2 macrophages, and other subsets (Figure S4H-K) and further annotated T cell subpopulations using “SingleR” 50. Both TLR2 and TLR4 were primarily expressed in macrophages, suggesting their potential roles in mediating immune responses within the TME (Figure 4B and Figure S4L). Interestingly, TLR2 was more highly expressed in M1 macrophages, whereas TLR4 expression was higher in M2 macrophages (Figure 4C and Figure S4M). Using “Network Analysis Toolkit for Multicellular Interactions” (NATMI) algorithm 51, we quantified intercellular communication within the TME and visualized interaction networks. Integrated analysis of GSE250521 and GSE193581 revealed that BGN⁺ cancer cells exhibited more frequent and stronger interactions with M2 macrophages among all TME cell types (Figure S4N-Q and Table S6). Ligand-receptor analysis further showed that BGN-TLR2 and BGN-TLR4 interactions occurred between BGN⁺ tumor or stromal cells and various myeloid cell subsets (Figure 4D). Importantly, BGN/TLR4 interactions showed higher expression and activity with M2 macrophages compared to BGN/TLR2 (Figure 4E and Figure S4R). It was suggested that BGN/TLR4 signaling may preferentially be involved in M2 polarization in PTC.

To assess the effect of BGN on macrophage migration, we co-cultured THP-1-derived macrophages with conditioned medium (CM) from BGN-knockdown or control KTC-1 and K1 cells. CM from BGN-knockdown cells significantly suppressed macrophage migration compared to controls (Figure 4F-G). Furthermore, when THP-1-derived macrophages were cultured with CM from BGN-knockdown cells for 48 h, qRT-PCR analysis showed downregulation of M2 macrophage markers (Arg1, CD163) and upregulation of M1 markers (CD86, CD11c) (Figure 4H). Flow cytometry confirmed that CM from BGN-knockdown cells significantly reduced the proportion of M2 macrophages (CD68⁺CD206⁺) and increased M1 macrophages (CD68⁺CD86⁺) (Figure 4I and Figure S4S-T). Similarly, CM from the BGN knockout group increased M1 proportions and reduced M2 proportions, whereas CM from BGN overexpression or supplementation with rBGN group reversed these effects (Figure 4J and Figure S4U).

To validate the role of TLR2/TLR4 in BGN-mediated M2 polarization, we generated BGN-overexpressing (OE-BGN) PTC cell lines from KTC-1 and K1 cells (Figure S4V). THP-1-derived macrophages were transfected with siRNA targeting TLR2 or TLR4 and co-cultured with CM from OE-BGN or control PTC cells for 48 h (Figure S4W-Y). CM from OE-BGN cells significantly upregulated M2 markers and downregulated M1 markers (Figure 4K). Importantly, TLR4 silencing—but not TLR2 silencing—significantly impaired BGN-induced M2 polarization (Figure 4K). Flow cytometry of M1 and M2 subsets further confirmed these findings (Figure 4L-M).

### Deposition of H3K27ac and BRD4 in the BGN enhancer and their interaction with the BGN promoter

**Figure 5:**
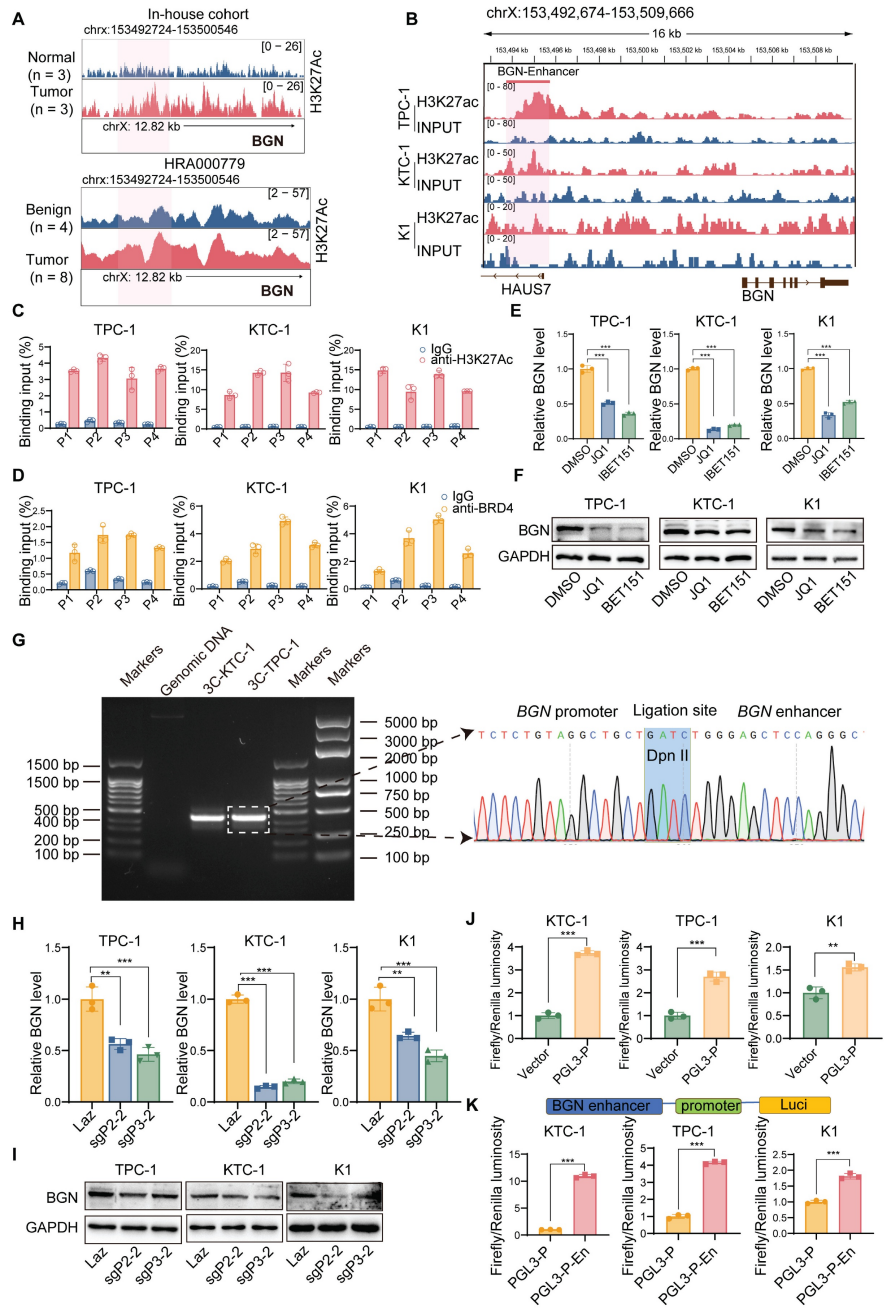
** BGN enhancer regulates its expression through epigenetic modifications. (A)** Peak plots showing the location of the BGN enhancer and the intensity of H3K27ac signals in this study (top) and previous studies (bottom). **(B)** Detection of the BGN enhancer in active enhancer regions marked by H3K27ac in PTC cell lines. **(C-D)** ChIP-qPCR analysis showing the enrichment of H3K27ac (C) and BRD4 (D) at the BGN enhancer region. **(E)** qRT-PCR analysis of BGN mRNA levels in PTC cells treated with BET inhibitors (JQ1 and I-BET151). **(F)** Western blot analysis of BGN protein levels in PTC cells treated with BET inhibitors (JQ1 and I-BET151). **(G)** Chromosome conformation capture (3C) assay confirming the interaction between the BGN enhancer and promoter and the junction sequence for 3C fragment was sequenced (right). **(H)** qRT-PCR analysis of BGN mRNA levels in PTC cells after CRISPRi-mediated inhibition of BGN enhancer activity. **(I)** Western blot analysis of BGN protein levels in PTC cells after CRISPRi-mediated inhibition of BGN enhancer activity. **(J)** Dual-luciferase reporter assay assessing the transcriptional activity of the BGN promoter. **(K)** Dual-luciferase reporter assay evaluating the regulatory effect of the BGN enhancer on the BGN promoter. (*p < 0.05, **p < 0.01, ***p < 0.001).

In our previous study, we performed ChIP-seq on H3K27ac in three pairs of PTC tumor tissues and adjacent normal tissues, identifying BGN as an enhancer-associated gene specific to PTC 52. Both our research center and the study by Zhang *et al*. 53 have revealed that the enhancer region of BGN is highly active in PTC tumor tissues, with significantly higher activity compared to adjacent normal or benign thyroid tissues (Figure 5A). To further elucidate the upstream regulatory mechanisms of BGN, we focused on exploring the regulatory network surrounding the enhancer-associated gene BGN.

Using ChIP-seq data from the ENCODE project, we analyzed histone modifications (H3K27ac, H3K4me1, and H3K4me3) in the BGN enhancer and its core promoter region 54. The results showed significant enrichment of H3K27ac and H3K4me1 modifications in the BGN enhancer region, while no prominent H3K4me3 peaks were detected (Figure S5A). Integration of H3K27ac modification data from PTC cell lines also revealed the presence of the BGN enhancer (Figure 5B). The BGN enhancer is located approximately 2 kb upstream of the BGN transcription start site. Considering that BRD4, an important transcriptional coactivator, plays a key role in the transcriptional activation of enhancer-associated genes 55, we designed four pairs of primers (P1, P2, P3, P4) and performed ChIP-qPCR to detect the binding of H3K27ac and BRD4 to the BGN enhancer region. The results showed that both H3K27ac and BRD4 were significantly enriched in the BGN enhancer region compared to the IgG control group (Figure 5C-D). When we treated PTC cells with BET inhibitors (JQ1 and I-BET151), these inhibitors blocked the binding of BET bromodomain proteins (such as BRD4) to H3K27ac 56. Experimental results demonstrated that both JQ1 and I-BET151 significantly downregulated BGN expression in PTC cells (Figure 5E-F). Additionally, ChIP-qPCR results revealed that JQ1 treatment also significantly reduced H3K27ac modification levels in the BGN enhancer region (Figure S5B).

Analysis of enhancer target genes using the GeneHancer database (ENCODE project) indicated that the BGN enhancer interacts with the adjacent BGN gene promoter 57 (Figure S5C). To confirm this interaction, we conducted the3C analysis. We found a 3C fragment formed by an upstream primer in the promoter region 252bp away from the DpnII restriction site, and a downstream primer in the promoter region 193bp away from DpnII, which revealed a significant physical interaction between the BGN enhancer and promoter (Figure 5G). Then, we employed an optimized CRISPR/Cas9 system, fusing a repressive KRAB domain with dCas9 (a Cas9 derivative with inactive enzymatic activity but retained DNA-binding specificity) and targeting the BGN enhancer with guide RNA 58 (Figure S5D). The results showed that inhibition of BGN enhancer activity significantly downregulated BGN expression in PTC cells (Figure 5H-I). ChIP-qPCR analysis further confirmed that after CRISPR interference of the BGN enhancer, H3K27ac and BRD4 enrichment at the P3 primer region was significantly reduced (Figure S5E).

A luciferase reporter plasmid, PGL3-P, containing the BGN promoter was conducted. The PGL3-P plasmid exhibited significantly higher dual-luciferase reporter activity compared to the control plasmid (Figure 5J). Based on this, we further constructed a luciferase reporter plasmid, PGL3-P-En, containing the BGN enhancer. In KTC-1, TPC-1, and K1 cells, the dual-luciferase reporter activity of the PGL3-P-En plasmid was significantly higher than that of the PGL3-P plasmid (Figure 5K). These findings collectively demonstrate that the BGN enhancer plays a crucial role in regulating BGN expression and suggest that enhancer-mediated transcriptional activation via BRD4 and H3K27ac modification is pivotal in PTC.

### NR2F2 regulates BGN transcription through enhancer activity, independent of CTCF-mediated chromatin looping

**Figure 6:**
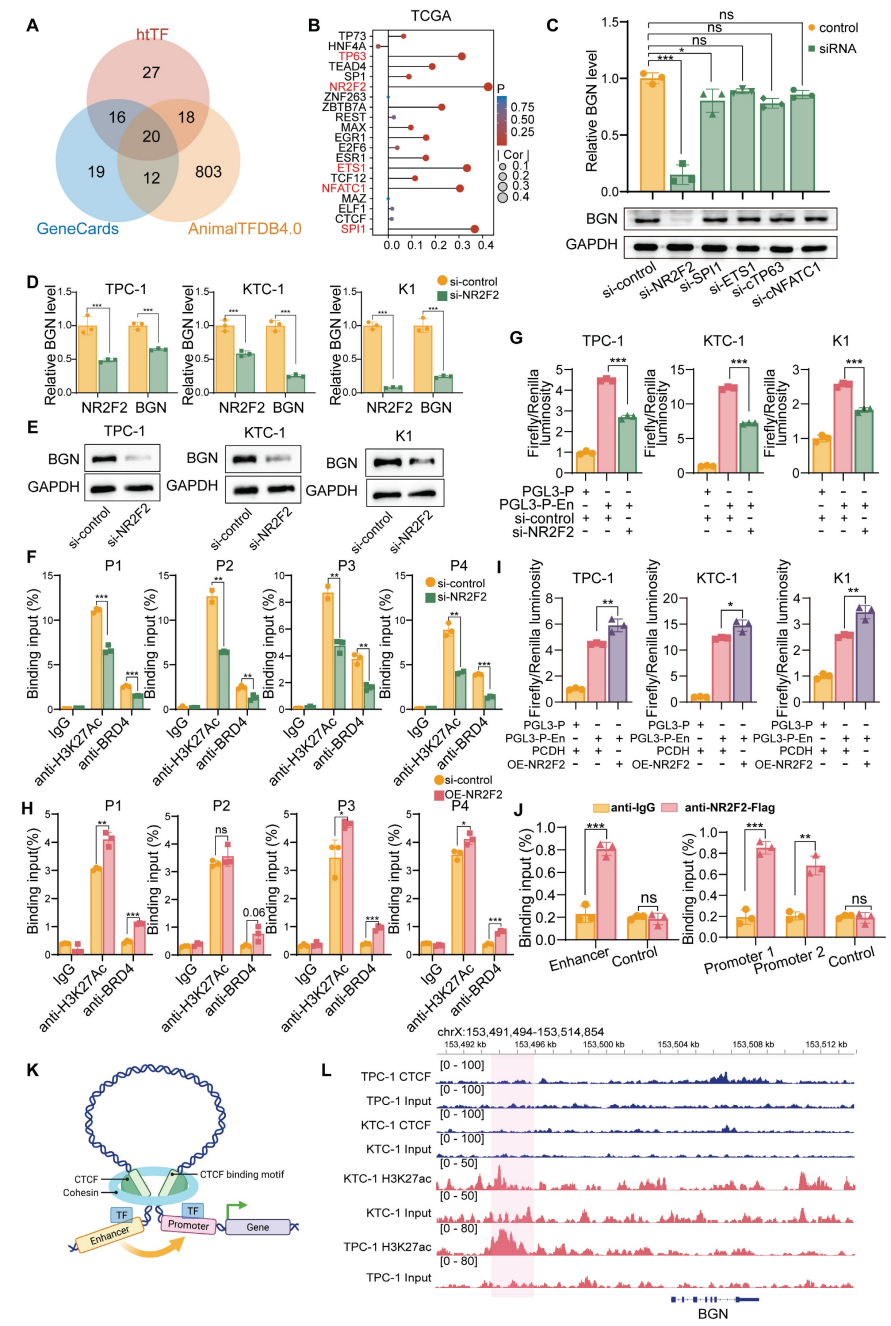
** NR2F2 regulates BGN transcription by modulating enhancer activity. (A)** There were 20 potential transcription factors regulating BGN identified from htTF, GeneCards, and AnimalTFDB4.0 databases. **(B)** Correlation analysis between transcription factors and BGN expression in the TCGA thyroid cancer dataset. **(C)** qRT-PCR and Western blot analysis of BGN expression following knockdown of transcription factors. **(D)** qRT-PCR analysis of NR2F2 and BGN mRNA levels in PTC cell lines after siRNA-mediated knockdown of NR2F2. **(E)** Western blot analysis of BGN expression following knockdown of transcription factors.** (F)** ChIP-qPCR analysis showing reduced H3K27ac and BRD4 levels at the BGN enhancer region following NR2F2 knockdown. **(G)** Dual-luciferase reporter assay evaluating the transcriptional activity of the BGN enhancer following NR2F2 knockdown. **(H)** ChIP-qPCR analysis of H3K27ac and BRD4 levels at the BGN enhancer region in PTC cell lines overexpressing NR2F2. **(I)** Dual-luciferase reporter assay assessing the transcriptional activity of the BGN enhancer following NR2F2 overexpression. **(J)** ChIP-qPCR analysis showing direct binding of NR2F2 to the BGN enhancer and promoter. **(K)** Diagram illustrating the role of CTCF in chromatin looping. (Created in BioRender. TAO, M. (2025) https://BioRender.com/yfl3u3w).** (L)** ChIP-seq tracks of CTCF binding at the BGN enhancer and promoter regions in KTC-1 and TPC-1 cells. (*p < 0.05, **p < 0.01, ***p < 0.001).

To elucidate the molecular mechanism by which the BGN enhancer regulates BGN transcription, we first screened 20 potential TFs associated with BGN from multiple transcription factor databases, including htTF, GeneCards, and AnimalTFDB4.0 (Figure 6A). We then performed a correlation analysis of the expression levels of these 20 TFs and BGN using the TCGA dataset, identifying the top five TFs that were positively correlated with BGN expression (Figure 6B). Further analysis of single-cell RNA-seq data in malignant PTC cells revealed that NR2F2 exhibited the strongest correlation with BGN expression (R = 0.341, *P* < 0.001) (Figure S6A).

To functionally assess the role of these TFs, we knocked down NR2F2, SPI1, ETS1, NFATC1, and TP63 using siRNA in the KTC-1 cell line (Figure S6B). Notably, NR2F2 knockdown significantly reduced BGN expression, whereas silencing SPI1, ETS1, NFATC1, or TP63 had no significant effect (Figure 6C).

Furthermore, RNA extraction and expression analysis in 34 PTC tissue samples revealed a significant positive correlation between NR2F2 and BGN expression (R = 0.607, *P* < 0.001) (Figure S6C). To explore the regulatory relationship between NR2F2 and the BGN enhancer, we performed motif analysis using the JASPAR database, which predicted NR2F2 binding sites in both the BGN enhancer and promoter regions (Figure S6D). Among these sites, the highest binding affinity was observed within the BGN enhancer region (Figure S6E and Table S8).

We then assessed the impact of NR2F2 depletion on BGN expression in PTC cell lines via siRNA-mediated knockdown. The results demonstrated that NR2F2 knockdown led to a significant reduction in BGN expression levels across all three cell lines (Figure 6D-E). ChIP-qPCR analysis further revealed that NR2F2 knockdown decreased H3K27ac and BRD4 enrichment at the BGN enhancer region (Figure 6F). Additionally, dual-luciferase reporter assay confirmed that NR2F2 depletion significantly inhibited BGN enhancer-mediated transcriptional activation (Figure 6G). We further established NR2F2-overexpressing stable cell lines using lentiviral transduction (OE-NR2F2) in PTC cells (Figure S6F-G). ChIP-qPCR analysis demonstrated that NR2F2 overexpression enhanced H3K27ac and BRD4 enrichment at the BGN enhancer region (Figure 6H). Furthermore, dual-luciferase reporter assay confirmed that NR2F2 overexpression significantly promoted BGN enhancer-driven transcriptional activation (Figure 6I). Finally, to directly assess NR2F2 binding to the BGN regulatory elements, we designed specific primers targeting the predicted NR2F2 binding sites within the BGN enhancer and promoter regions for ChIP assays. The results confirmed that NR2F2 directly binds to both the BGN enhancer and promoter (Figure 6J).

However, the role of CTCF, a well-known factor in chromatin looping, in regulating BGN transcription was also examined, as it has been shown to facilitate enhancer-promoter interactions through chromatin looping (Figure 6K) 59. We performed CTCF ChIP-seq in KTC-1 and TPC-1 cells to assess whether CTCF plays a role in BGN enhancer regulation (Figure S6H). The results indicated that CTCF does not bind to the BGN enhancer or promoter regions (Figure 6L). ChIP-seq data further revealed that CTCF binding was enriched around other gene promoters, such as PLXNB2, but not near the BGN gene (Figure S6I-J). In conclusion, our findings establish NR2F2 as a key regulator of BGN transcription in PTC. By binding to the BGN enhancer, NR2F2 promotes transcriptional activation by facilitating the enrichment of H3K27ac and BRD4 at the enhancer. Notably, CTCF-mediated chromatin looping does not appear to play a role in this process, as no CTCF binding was observed in the regions surrounding the BGN enhancer and promoter.

### The NR2F2-BGN axis promotes PTC progression via AKT signaling and macrophage polarization

**Figure 7:**
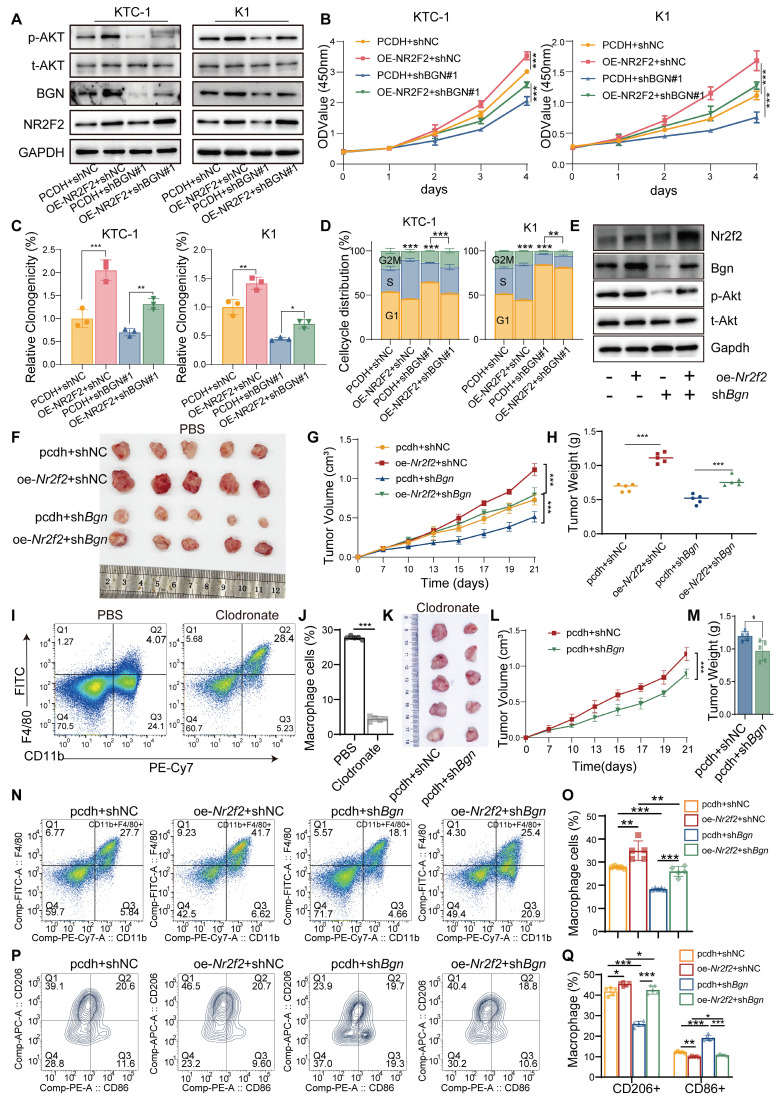
** NR2F2-BGN axis promotes PTC progression via AKT signaling and macrophage polarization. (A)** Western blot analysis of p-AKT, t-AKT, NR2F2, and BGN expression in PTC cell lines with NR2F2 overexpression and BGN knockdown. **(B)** CCK-8 assay assessing the impact of the NR2F2-BGN axis on cell viability in KTC-1 and K1 cell lines. **(C)** Colony formation assays evaluating the effect of the NR2F2-BGN axis on cell proliferation in KTC-1 and K1 cell lines. **(D)** Flow cytometry analysis of cell cycle distribution in KTC-1 and K1 cell lines following NR2F2-BGN axis modulation. **(E)** Western blot analysis of p-Akt, t-Akt, Nr2f2, and Bgn expression in murine-derived thyroid cancer with Nr2f2 overexpression and Bgn knockdown.** (F)** Subcutaneous tumor of murine thyroid cancer cells from C57BL/6J mice treated with PBS liposomes in different experimental groups. **(G)** Tumor growth curves illustrating the effect of the Nr2f2-Bgn axis on murine subcutaneous tumor growth in C57BL/6J mice. **(H)** Tumor weight comparison demonstrating the impact of the Nr2f2-Bgn axis on murine subcutaneous tumor burden. **(I)** Representative flow cytometry chart demonstrating macrophage depletion following treatment with clodronate liposomes and PBS liposomes.** (J)** Flow cytometry analysis of macrophages infiltration in murine tumors after using clodronate liposomes.** (K)** Subcutaneous tumor of murine thyroid cancer cells from C57BL/6J mice treated with clodronate liposomes in in control and Bgn knockdown groups.** (L)** Tumor growth curves of murine thyroid cancer cells from C57BL/6J mice treated with clodronate liposomes in control and Bgn knockdown groups.** (M)** Tumor weight of murine thyroid cancer cells from C57BL/6J mice treated with clodronate liposomes in control and Bgn knockdown groups.** (N)** Representative flow cytometry plots showing the proportion of macrophages infiltration (CD45^+^CD11b^+^F4/80^+^) in different groups.** (O)** Flow cytometry analysis of the proportion of macrophages infiltration (CD45^+^CD11b^+^F4/80^+^) in different groups.** (P)** Representative flow cytometry plots showing the proportion of M1 (CD86⁺CD206^-^) and M2 macrophages (CD86^-^CD206^+^) in different groups. **(Q)** Flow cytometry analysis of the proportion of M1 (CD86⁺CD206^-^) and M2 macrophages (CD86^-^CD206^+^) in different groups. (*p < 0.05, **p < 0.01, ***p < 0.001).

Based on the aforementioned findings, NR2F2 was shown to positively regulate BGN expression at the transcriptional level by directly binding to the BGN enhancer and promoter regions. This suggests that the NR2F2-BGN axis plays a pivotal role in the pathogenesis of Papillary Thyroid Cancer (PTC). To further investigate its biological function, we established four experimental groups: control (PCDH+shNC), NR2F2 overexpression (OE-NR2F2+shNC), BGN knockdown (PCDH+ shBGN#1), and BGN knockdown with NR2F2 overexpression (OE-NR2F2+ shBGN#1). Functional analysis demonstrated that NR2F2 overexpression significantly activated the AKT signaling pathway and rescued the inhibition of the AKT pathway caused by BGN knockdown (Figure 7A). Further functional assays revealed that NR2F2 overexpression partially restored the loss of cell viability and colony formation ability induced by BGN knockdown in PTC cells (Figure 7B-C and Figure S7A). Cell cycle analysis showed that NR2F2 overexpression alleviated G1-phase arrest caused by BGN depletion (Figure 7D and Figure S7B).

Additionally, we established a murine thyroid cancer cell line model using the same experimental groups: control (pcdh+shNC), Nr2f2 overexpression (oe-Nr2f2+shNC), Bgn knockdown (pcdh+shBgn), and Bgn knockdown with Nr2f2 overexpression (oe-Nr2f2+shBgn). The results indicated that the effects observed in the murine thyroid cancer cell lines were consistent with those in human PTC cells (Figure 7E). We implanted these cells into C57BL/6J mice, and tumor growth was monitored regularly.

Tumors were harvested for imaging and analysis once significant differences between groups were observed (Figure 7F). Tumor growth curves and tumor weight measurements revealed that Nr2f2 overexpression significantly promoted tumor growth, compared to the control group. Furthermore, BGN knockdown partially reduced the tumor growth promotion caused by Nr2f2 overexpression (Figure 7G-H). To further investigate the role of immune cells in this context, we utilized Clodronate liposome treatment to deplete macrophages in the tumors (Figure 7I-J). After macrophage depletion, the inhibitory effect of Bgn knockdown on tumor growth was significantly reduced, indicating that in an immunocompetent model, both Bgn and macrophages play crucial roles in promoting tumor progression (Figure 7K-M). Additionally, we established a human macrophage-tumor cell co-injection model in nude mice, where PTC tumor cells and THP-1 cells were injected subcutaneously at a 5:1 ratio 35. Tumor growth was monitored, and tumors were harvested for analysis when significant differences between groups were observed (Figure S7C). Tumor growth curves and tumor weight measurements showed that NR2F2 overexpression significantly promoted tumor growth, while BGN knockdown partially inhibited the tumor growth promotion caused by NR2F2 overexpression (Figure S7D-E). Immunohistochemical analysis of tumor tissues further revealed a positive correlation between Ki67 and p-AKT expression levels and NR2F2-BGN axis activity (Figure S7F-H).

Flow cytometry analysis of macrophage infiltration in mouse tumor tissues revealed that the Nr2f2-Bgn axis altered the proportion of tumor-associated macrophages (TAMs) (Figure S7J). Overexpression of Nr2f2 promoted macrophage infiltration, whereas Bgn knockdown inhibited macrophage infiltration. In the Nr2f2 overexpression group, Bgn knockdown reversed the raise in macrophage infiltration (Figure 7N-O). Furthermore, Nr2f2 overexpression increased the proportion of M2-like macrophages, while decreasing the proportion of M1-like macrophages. Bgn knockdown had the opposite effect, increasing M1 macrophages and decreasing M2 macrophages. Notably, in the Nr2f2 overexpression group, BGN knockdown reversed the increase in M2 macrophage proportion (Figure 7P-Q). These results suggest that the Nr2f2-Bgn axis influences both macrophage infiltration and polarization in the tumor microenvironment. We also examined macrophage polarization in the human macrophage-tumor cell co-injection model in nude mice. NR2F2 overexpression significantly decreased the proportion of M1-like macrophages while promoting M2-like macrophage polarization compared to the control group. Additionally, BGN knockdown partially reversed the increase in M2-like macrophages and the decrease in M2-like macrophages caused by NR2F2 overexpression (Figure S7K-M). These findings provide compelling evidence that the NR2F2-BGN axis plays a crucial role in PTC progression by activating AKT signaling and promoting M2 macrophage polarization. This highlights the NR2F2-BGN axis as a promising therapeutic target for PTC.

### NR2F2 inhibitor CIA1 inhibits thyroid cancer progression and modulates the tumor immune microenvironment through targeting the NR2F2-BGN axis

**Figure 8:**
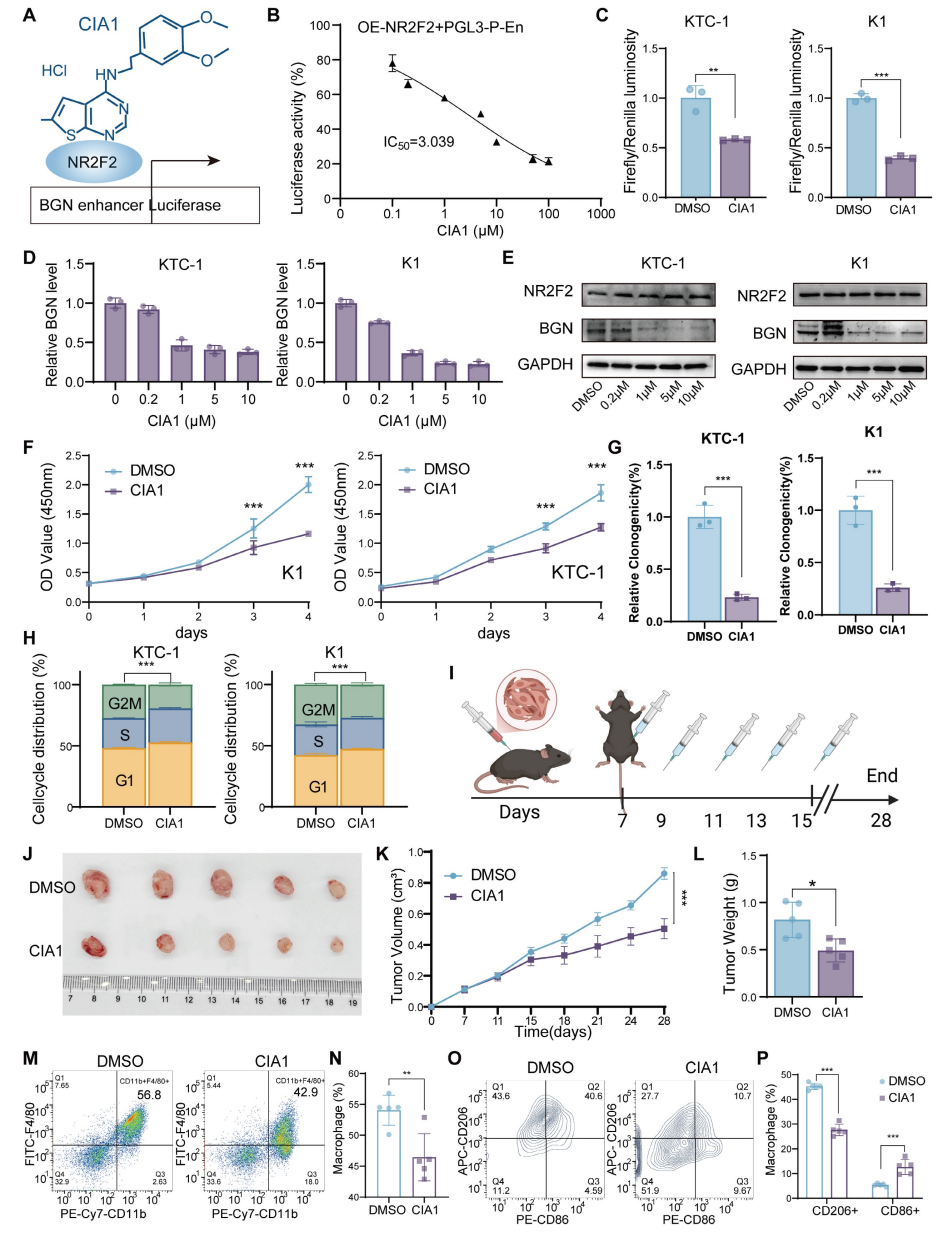
** Pharmacological inhibition of NR2F2 with CIA1 suppresses thyroid cancer progression and modulates tumor immune microenvironment. (A)** Dual-luciferase assay in 293T cells transfected with NR2F2 expression plasmid and treated with CIA1 for 24 hours to measure luciferase activity. **(B)** CIA1 dose-dependently inhibited NR2F2-mediated luciferase activity driven by the BGN promoter. **(C)** In KTC-1 and K1 cells co-transfected with NR2F2 and BGN-enhancer constructs, 5 μM CIA1 significantly reduced luciferase activity. **(D)** qRT-PCR analysis showing that CIA1 treatment significantly reduced BGN mRNA levels in KTC-1 and K1 cells. **(E)** Western blot showing that CIA1 dose-dependently decreased NR2F2 and BGN protein levels. **(F)** CCK-8 assay showing that CIA1 treatment inhibited the proliferation of KTC-1 and K1 cells. **(G)** Colony formation assays further confirmed that CIA1 suppressed clonogenic capacity in KTC-1 and K1 cells. **(H)** Flow cytometry analysis showing that CIA1 treatment induced G1-phase cell cycle arrest in KTC-1 and K1 cells. **(I)** Schematic of CIA1 administration in C57BL/6J mice with subcutaneously implanted thyroid cancer cells. (Created in BioRender. TAO, M. (2025) https://BioRender.com/t5za510). **(J)** Representative images showing tumor growth and tumor tissue collection. **(K)** CIA1 treatment resulted in a significant reduction in tumor weight. **(L)** The tumor growth curve of CIA1-treated mice compared to DMSO-treated controls. **(M-N)** Representative images (M) and statistical comparison (N) of macrophage infiltration in tumors from DMSO vs CIA1-treated mice. **(O-P)** Representative images (O) and statistical analysis (P) of the M1/M2 macrophages polarization proportion in CIA1 vs DMSO-treated tumors. (*p < 0.05, **p < 0.01, ***p < 0.001).

Recent studies have reported the development of CIA1, a specific inhibitor of NR2F2 (COUP-TFII), which targets the ligand-binding domain of NR2F2 to disrupt its interaction with transcription factors like FOXA1. This prevents NR2F2 from regulating downstream target genes and has been shown to effectively inhibit prostate cancer growth 60. In previous work, it was demonstrated that NR2F2 can bind to and activate the BGN promoter and enhancer regions, promoting its expression and facilitating tumor progression and the formation of an immunosuppressive microenvironment. To investigate the mechanism of CIA1 in the NR2F2-BGN axis, we first established a dual-luciferase reporter assay in 293T cells to evaluate the transcriptional activity of NR2F2 at the BGN enhancer following CIA1 treatment (Figure 8A). The results showed that CIA1 significantly suppressed the NR2F2-mediated activation of the BGN enhancer, with a dose-dependent inhibition (Figure 8B).

This effect was similarly observed in the PTC cell lines KTC-1 and K1, where CIA1 reduced NR2F2-driven BGN enhancer activity (Figure 8C). The results of hematological and biochemical analyses indicate that CIA1 treatment does not affect liver or kidney function, nor does it alter routine blood parameters in mice, thereby supporting the safety of CIA1 (Table S9-S10). qRT-PCR and Western blot analyses confirmed that CIA1 treatment significantly decreased both BGN mRNA and protein levels, while NR2F2 expression remained unaffected (Figure 8D-E), suggesting that CIA1's inhibitory effect is primarily due to disruption of NR2F2's transcriptional function.

Functional assays demonstrated that CIA1 significantly inhibited the proliferation and clonogenic ability of KTC-1 and K1 cells (Figure 8F-G and Figure S8A), and induced G1-phase cell cycle arrest (Figure 8H and Figure S8B). In the subcutaneous murine thyroid cancer model in C57BL/6J mice, intraperitoneal injection of CIA1 (5 mg/kg) significantly inhibited tumor growth, as evidenced by reduced tumor weight and slower tumor volume progression (Figure 8I-L). HE staining showed no significant toxicity in major organs (Figure S8C), indicating good systemic tolerability of CIA1. Immunohistochemical analysis of tumor tissues demonstrated that CIA1 treatment downregulated BGN, Ki67, and p-AKT expression, while NR2F2 and total AKT levels remained unchanged (Figure S8D-E), suggesting that CIA1 primarily acts through inhibition of the NR2F2-BGN axis.

Additionally, flow cytometry analysis of tumor immune cell composition (Figure S8F) revealed that CIA1 treatment significantly reduced the proportion of tumor-infiltrating macrophages (CD45⁺CD11b⁺F4/80⁺) (Figure 8M-N) and altered their polarization state: the proportion of M2-type macrophages (CD86⁻CD206⁺) was decreased, while M1-type macrophages (CD86⁺CD206⁻) increased (Figure 8O-P). This suggests that CIA1 may regulate macrophage polarization and the tumor immune microenvironment through targeting the NR2F2-BGN axis, providing a potential foundation for its therapeutic application in PTC.

### The NR2F2-BGN expression in PTC was positively correlated with M2 macrophage infiltration and associated with clinical features

**Figure 9:**
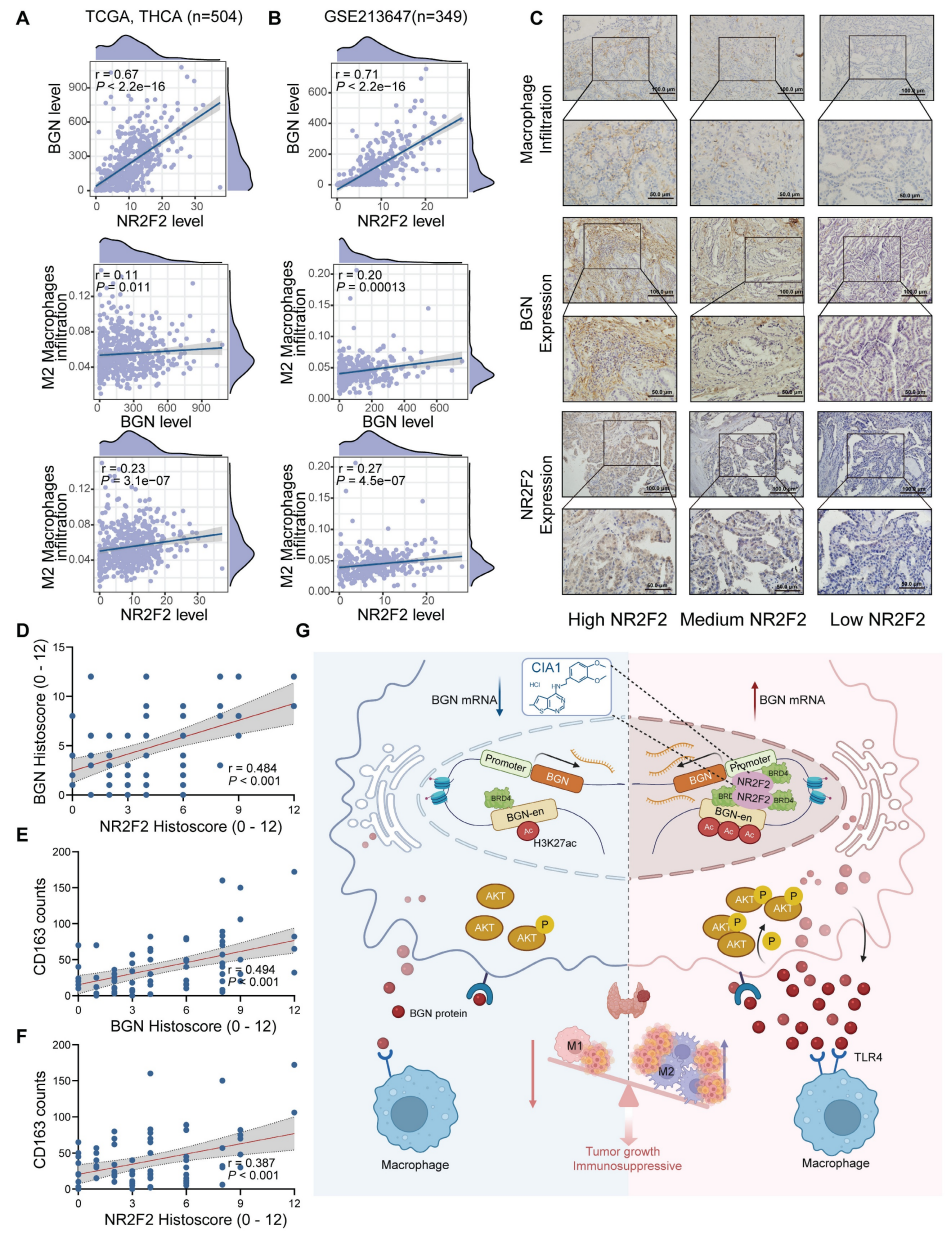
** Correlation between NR2F2, BGN expression, and M2 macrophage infiltration in PTC. (A-B)** Correlation analysis of NR2F2 and BGN expression in malignant cells, as determined by BayesPrism deconvolution, with macrophage infiltration in the TCGA (A) and GSE213647 (B) thyroid cancer dataset. **(C)** Representative immunohistochemical images of NR2F2, BGN, and CD163 staining in PTC tissue samples. **(D-F)** Correlation analysis of NR2F2 and BGN protein expression with CD163-positive macrophage infiltration. **(G)** This schematic illustrates the mechanism by which NR2F2 promotes BGN transcription and tumor progression in PTC. (Created in BioRender. TAO, M. (2025) https://BioRender.com/0967543). (*p < 0.05, **p < 0.01, ***p < 0.001).

To investigate the relationship between NR2F2, BGN, and M2 macrophages, we integrated bulk RNA-seq data with IHC staining results from PTC patients in our center to analyze their expression correlation and clinical significance in PTC tissues. The BayesPrism analysis of 504 PTC samples from the TCGA database and 349 PTC samples from the GSE213647 dataset revealed a significant positive correlation between NR2F2 and BGN mRNA expression levels in malignant cells (TCGA: R = 0.67, *P <* 0.001; GSE213647: R = 0.71, *P <* 0.001). Furthermore, BGN mRNA expression was positively correlated with M2 macrophage infiltration (TCGA: R = 0.11, *P* = 0.011; GSE213647: R = 0.20, *P <* 0.001), and NR2F2 mRNA expression similarly exhibited a positive correlation with M2 macrophage infiltration (TCGA: R = 0.23, *P* < 0.001; GSE213647: R = 0.27, *P* < 0.001) (Figure 9A-B).

We performed IHC staining on 71 PTC tissue samples to assess the correlation between NR2F2 and BGN protein expression and macrophage infiltration (Figure 9C). Consistent with the RNA-seq data, NR2F2 protein expression was significantly correlated with BGN protein expression (R = 0.484, *P <* 0.001) (Figure 9D). M2 macrophages were identified by CD163 staining, and quantification of CD163+ cells within the tumor microenvironment demonstrated a significant positive correlation between BGN protein expression and M2 macrophage infiltration (R = 0.494, *P <* 0.001) (Figure 9E). Similarly, NR2F2 protein expression was positively associated with CD163+ macrophage infiltration (R = 0.387, *P <* 0.001) (Figure 9F).

To further investigate the clinical significance of NR2F2 and BGN expression, we stratified PTC patients into two groups based on IHC scores: the NR2F2^high^BGN^high^group (NR2F2 IHC score ≥ 6 and BGN IHC score≥ 6) and the other group. Clinicopathological analysis revealed that the NR2F2^high^BGN^high^ group was significantly associated with larger tumor size (*χ²* = 4.978, *P =* 0.026) and more advanced pathological T stage (*χ²* = 9.953, *P =* 0.002) (Table 1). These findings, at both the transcriptomic and proteomic levels, highlight a strong association between NR2F2, BGN, and M2 macrophages in PTC. Furthermore, they underscore the potential clinical relevance of the NR2F2^high^BGN^high^ phenotype in PTC progression.

## Discussion

This study revealed the mechanism by which NR2F2 promotes BGN transcription and tumor progression in PTC. NR2F2 activates the BGN gene by binding to its enhancer (BGN-en) and promoter regions, recruiting the transcriptional coactivator BRD4 and enhancing H3K27ac histone modifications. This results in chromatin looping and increased BGN expression, which in turn activates the AKT signaling pathway, promoting tumor cell proliferation, migration, and invasion. BGN also acts in a paracrine manner via TLR4 receptors on macrophages to induce M2 macrophage polarization, contributing to an immunosuppressive tumor microenvironment. Treatment with the NR2F2 inhibitor CIA1 blocks NR2F2 activation of BGN, reducing BGN expression and AKT pathway activity, inhibiting malignant behavior of tumor cells, and reversing macrophage polarization, thereby restoring antitumor immunity. These findings highlight the NR2F2-BGN axis as a potential therapeutic target in PTC. (Figure 9G).

Endogenous molecules released from the ECM can serve as DAMPs and are critical regulators of macrophage activation 39, 40, 61. Studies have shown that tumor-secreted proteoglycans activate the TLR2/TLR6 complex on macrophages, utilizing the body's innate immune system for immune evasion 62. In inflammation-related diseases, BGN has been extensively studied as a natural ligand for TLR2/TLR4 receptors and an important pro-inflammatory factor 47-49. However, some studies also suggest that BGN exerts anti-inflammatory effects by inhibiting IL-1β production 63. Interestingly, our study found that BGN secreted by PTC tumor cells drives M2 macrophage polarization through the TLR4 signaling pathway, thereby exerting an anti-inflammatory effect. We hypothesize that during PTC progression, secreted BGN protein may engage additional co-receptor molecules when binding to TLR4 on macrophages. In the BGN and TLR2/TLR4 signaling pathways, CD14 acts as a co-receptor in the sterile inflammatory response induced by renal ischemia/reperfusion injury in mice, and its deficiency significantly inhibits macrophage recruitment 64. In contrast, during early renal ischemia/reperfusion injury, CD44, as a co-receptor for the BGN/TLR4 signaling pathway, induces autophagy in M1 macrophages, which in turn enhances M2 macrophage polarization. Based on these findings, future research will focus on the role of these co-receptor molecules to further elucidate the specific mechanisms of BGN/TLR4 signaling in reshaping the immune microenvironment in PTC.

We also sought to delineate the relative contribution of BGN's tumor-intrinsic effects versus its immunomodulatory function. In immune-competent C57BL/6J mice, macrophage depletion markedly attenuated the tumor-suppressive effects observed upon BGN knockdown, suggesting that BGN's role in shaping the immune microenvironment is critical for its tumor-promoting activity. In contrast, in immunodeficient nude mice, BGN knockdown still significantly reduced tumor growth, albeit to a lesser extent. These findings collectively indicate that BGN functions through dual mechanisms, with its immunomodulatory role via macrophage polarization being dominant in immune-competent contexts. Therefore, while BGN directly enhances tumor cell aggressiveness, its impact on immune suppression appears to be a major driver of tumor progression *in vivo*. GABA derived from tumor cells promotes lung cancer progression, not by directly affecting cancer cells, but by influencing macrophage polarization in the tumor microenvironment 65. Macrophages are also essential in arginine synthesis inhibition-mediated breast cancer growth suppression 66. In the tumor microenvironment, we cannot ignore the influence of any single factor; these factors interact with each other, promoting and restraining each other. Furthermore, the model in nude mice also revealed the tumor-promoting effect of BGN secreted by tumor cells, which may rely on pro-cancer signals mediated by integrins expressed on the tumor cells, subsequently activating the AKT signaling pathway and promoting tumor progression via the ECM-cell interactions 67, 68.

Although previous studies have suggested that alterations in certain signaling pathways may lead to BGN upregulation, direct evidence of its transcriptional regulatory mechanisms remains lacking 8, 41. Here, we identified an enhancer that regulates BGN expression and revealed its molecular mechanism of transcriptional activation via the recruitment of the transcription factor NR2F2 to interact with the BGN promoter. Promoter-enhancer interactions are a core mechanism in gene transcription regulation 69. In the chromatin looping model, gene expression is regulated through the coordinated actions of DNA-binding proteins and protein complexes, such as transcription factors, coactivators, corepressors, and RNA polymerase II. These protein complexes regulate chromatin accessibility and activity by binding to DNA sequences in the promoter and enhancer regions, thereby influencing gene transcription 70. In this study, we used 3C experiments to demonstrate the physical interaction between the BGN enhancer and promoter, further validating this model.

BRD4, a key member of the BET family, acts as a coactivator in the chromatin looping model, binding to acetylated histones like H3K27ac and enriching transcriptionally active regulatory elements to promote the initiation and elongation of gene transcription 71, 72. We discovered that the BGN enhancer region is significantly enriched with BRD4 and H3K27ac modifications. Treatment with BRD4 inhibitors (such as JQ1 and I-BET151) significantly reduced BGN expression levels, suggesting that BRD4 inhibition may represent a potential therapeutic strategy to disrupt the oncogenic effects of the BGN enhancer.

NR2F2, a member of the nuclear receptor (NR) family, is classified as an orphan receptor due to the lack of known natural ligands 73. NR2F2 is highly expressed during embryonic differentiation and in adult mesenchymal cells, where it regulates cell metabolism and differentiation 74, 75. In cancer, the role of NR2F2 is controversial, as it may act as both an oncogene and a tumor suppressor gene. NR2F2 is uniquely expressed in malignant squamous cell carcinoma, promoting tumor stemness and invasiveness while inhibiting differentiation, thereby maintaining the tumor's malignant state 76. In Luminal A breast cancer, high NR2F2 expression is associated with improved patient survival 77. We found that NR2F2 directly binds to both the BGN enhancer and promoter regions, activating BGN transcription, thereby driving PTC tumor progression and the formation of an immunosuppressive microenvironment. We also utilized the previously reported NR2F2-specific inhibitor CIA1 60. This therapy had surprising and promising results in our thyroid cancer study, further confirming the potential clinical value of targeting the NR2F2-BGN axis.

To further dissect the transcriptional regulation of BGN, we investigated the architectural protein CTCF, known for its role in chromatin looping. Together with the cohesin complex or colocalizing with TFs, CTCF establishes chromatin loops and topologically associated domains to regulate gene expression by facilitating enhancer-promoter interactions 78. Interestingly, our data suggest that CTCF is not involved in bridging the BGN enhancer and promoter. This implies alternative looping mediators may be at play. Most enhancer-promoter interactions do not rely on CTCF-CTCF interactions, and other architectural proteins, such as YY1, can regulate enhancer-promoter loops independent of CTCF 79. Further studies exploring the involvement of YY1 or other architectural proteins are warranted to clarify the precise regulatory architecture of the BGN locus.

Our findings also bear broader implications beyond PTC. BGN is widely implicated in various malignancies and has been shown to influence immune cell function and therapeutic response in several cancers. In breast cancer, BGN secreted by CAFs negatively correlates with CD8+ T cell infiltration and promotes immune evasion 37. Bgn knockout in murine models enhances CD8+ T cell infiltration and sensitizes tumors to chemotherapy 80. These observations suggest that the BGN-mediated immunosuppressive microenvironment may be a common feature across tumor types. Moreover, BGN's activation of innate immune receptors such as TLR2 and TLR4 further underscores its potential as a universal modulator of tumor immunity. Thus, the conclusions of this study may be extrapolated beyond PTC, particularly in cancers where the tumor associated macrophages play a pivotal role. BGN has also been described as a natural ligand for TLR2/TLR4 and an important pro-inflammatory molecule 47-49. However, we observed that in the context of PTC, BGN promotes M2 polarization of macrophages, which is typically associated with anti-inflammatory and immunosuppressive states. This apparent paradox may be explained by the involvement of co-receptors. For instance, CD14 and CD44 have been identified as co-receptors in the BGN-TLR4 pathway in renal injury models, modulating macrophage recruitment and polarization 64. We hypothesize that in PTC, similar co-receptors may cooperate with TLR4 to mediate the immunosuppressive effects of BGN, a hypothesis that warrants further investigation.

As genomic research expands from exome sequencing to whole-genome analysis, our understanding of regulatory mechanisms in non-coding regions continues to deepen. Regulatory elements such as enhancers play crucial roles in disease development, but their specific functional mechanisms remain largely unexplored. This study demonstrates that the BGN enhancer regulates BGN expression by recruiting the transcription factor NR2F2, shedding light on the key role of non-coding regions in PTC and providing new clues for exploring enhancer-mediated gene regulatory networks. Future research should focus on targeting the BGN enhancer-NR2F2-BGN axis, particularly in combination with immunotherapy.

## Conclusion

In conclusion, our study not only reveals a novel enhancer-mediated transcriptional mechanism involving NR2F2 and BRD4 that activates BGN in PTC but also highlights BGN's dual role in promoting tumor cell-intrinsic aggressiveness and shaping the tumor immune environment. These findings open new avenues for targeting the NR2F2-BGN axis, both in PTC and potentially in other malignancies where BGN plays a central role in tumor progression and immune modulation. Furthermore, the use of the NR2F2-specific inhibitor CIA1 in our study shows promising therapeutic potential, further supporting the clinical value of targeting the NR2F2-BGN axis in cancer treatment.

## Supplementary Material

Supplementary figures and tables.

## Figures and Tables

**Table 1:** The association of NR2F2 and BGN levels with clinicopathological indicators in papillary thyroid carcinoma tissues.

Characteristics	Total	NR2F2^high^BGN^high^	Others	χ^2^	P value
n	71	17	54		
**Gender**				0	1.000
Female	56	13 (18.3%)	43 (60.6%)		
Male	15	4 (5.6%)	11 (15.5%)		
**Age**				<0.001	1.000
<55 years	55	13 (18.3%)	42 (59.2%)		
≥55 years	16	4 (5.6%)	12 (16.9%)		
**Tumor size (cm)**				4.978	0.023^*^
≤1	29	9 (12.7%)	49 (69%)		
>1	42	8 (11.3%)	5 (7%)		
**pT stage**				9.953	0.002^ *^
pT1/2	58	37 (52.1%)	21 (29.6%)		
pT3/4	13	3 (4.2%)	10 (14.1%)		
**pN stage**				5.129	0.077
pN0	34	5 (7%)	29 (40.8%)		
pN1a	24	6 (8.5%)	18 (25.4%)		
pN1b	13	6 (8.5%)	7 (9.9%)		
**BRAF**				2.220	0.136
Wild	23	3 (4.2%)	20 (28.2%)		
Mutation	48	14 (19.7%)	34 (47.9%)		
**Multifocality**				0.212	0.646
No	41	9 (12.7%)	32 (45.1%)		
Yes	30	8 (11.3%)	22 (31%)		
**Hashimoto thyroiditis**				1.054	0.305
No	47	24 (33.8%)	23 (32.4%)		
Yes	24	16 (22.5%)	8 (11.3%)		

## Abbreviations

TFs: Transcription factors
GEO: Gene Expression Omnibus
ATC: Anaplastic thyroid carcinoma
TCGA: The Cancer Genome Atlas
FBS: Fetal bovine serum
CM: Conditioned medium
3C: Chromosome Conformation Capture
CRISPRi: CRISPR interference
IHC: Immunohistochemical
OS: Overall survival
PFS: Progression-free survival
DFS: Disease-free survival
rBGN: Recombinant BGN protein
scRNA-seq: Single-cell RNA sequencing
spRNA-seq: Spatial RNA sequencing
DEGs: Differentially expressed genes
CTSK: Cathepsin K
CAFs: Cancer-associated fibroblasts
DAMPs: Damage-associated molecular patterns
NR: Nuclear receptor
SMCs: Smooth muscle cells
TFs: Transcription factors
GEO: Gene Expression Omnibus
ATC: Anaplastic thyroid carcinoma
TCGA: The Cancer Genome Atlas
