# Supplementary material for: Enhancer-mediated NR2F2 recruitment activates BGN to promote tumor growth and shape tumor microenvironment in papillary thyroid cancer
Source: Theranostics. 2026 Jan 1;16(1):298–324. doi: 10.7150/thno.113712 (PMC12665125; doi:10.7150/thno.113712)
Supplement: Supplementary file 1 — Supplementary figures and tables. [file thnov16p0298s1.zip › Supplementary figures and tables.pdf]

Enhancer-mediated NR2F2 recruitment activates BGN to promote tumor growth and shape tumor microenvironment in papillary thyroid cancer

Mei Tao, Xianhui Ruan, Jialong Yu, Qiman Dong, Wei Luo, Wei Zhang, Mengran Tian, Xiukun Hou, Linfei Hu, Jingzhu Zhao, Dapeng Li, Jie Hao, Songfeng Wei, Xiangqian Zheng, Ming Gao

Figure S1

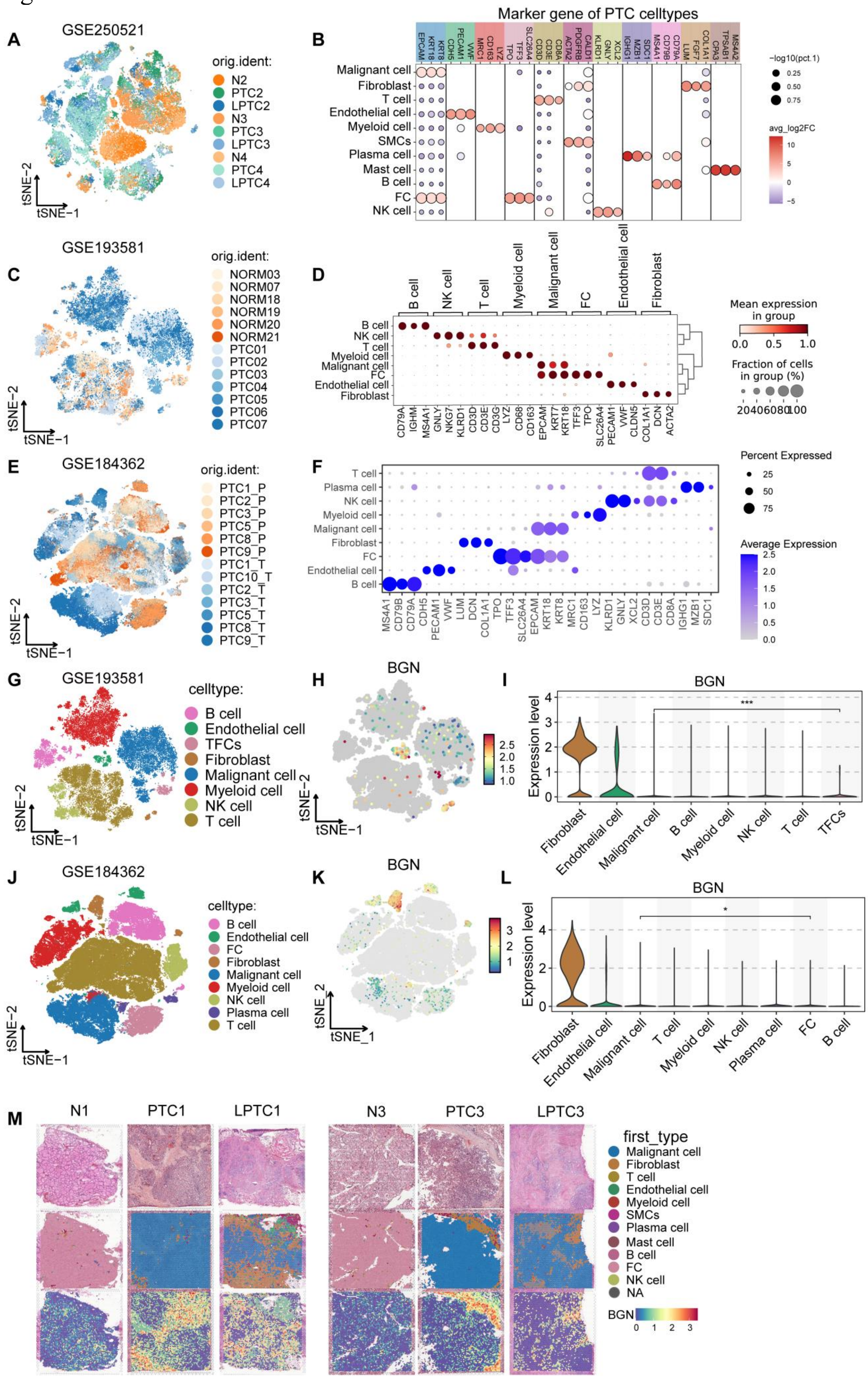
A
GSE250521
orig.ident:
N2
PTC2
LPTC2
N3
PTC3
LPTC3
N4
PTC4
LPTC4
tSNE-1
tSNE-2
B
Marker gene of PTC celltypes
Malignant cell
Fibroblast
T cell
Endothelial cell
Myeloid cell
SMCs
Plasma cell
Mast cell
B cell
FC
NK cell
-log10(pct.1)
avg_log2FC
C
GSE193581
D
E
GSE184362
F
Percent Expressed
Average Expression
Mean expression in group
Fraction of cells in group (%)
G
celltype:
B cell
Endothelial cell
TFCs
Fibroblast
Malignant cell
Myeloid cell
NK cell
T cell
H
BGN
I
Expression level
J
K
L
M
N1
PTC1
LPTC1
N3
PTC3
LPTC3
first_type
Malignant cell
Fibroblast
T cell
Endothelial cell
Myeloid cell
SMCs
Plasma cell
Mast cell
B cell
FC
NK cell
NA
BGN

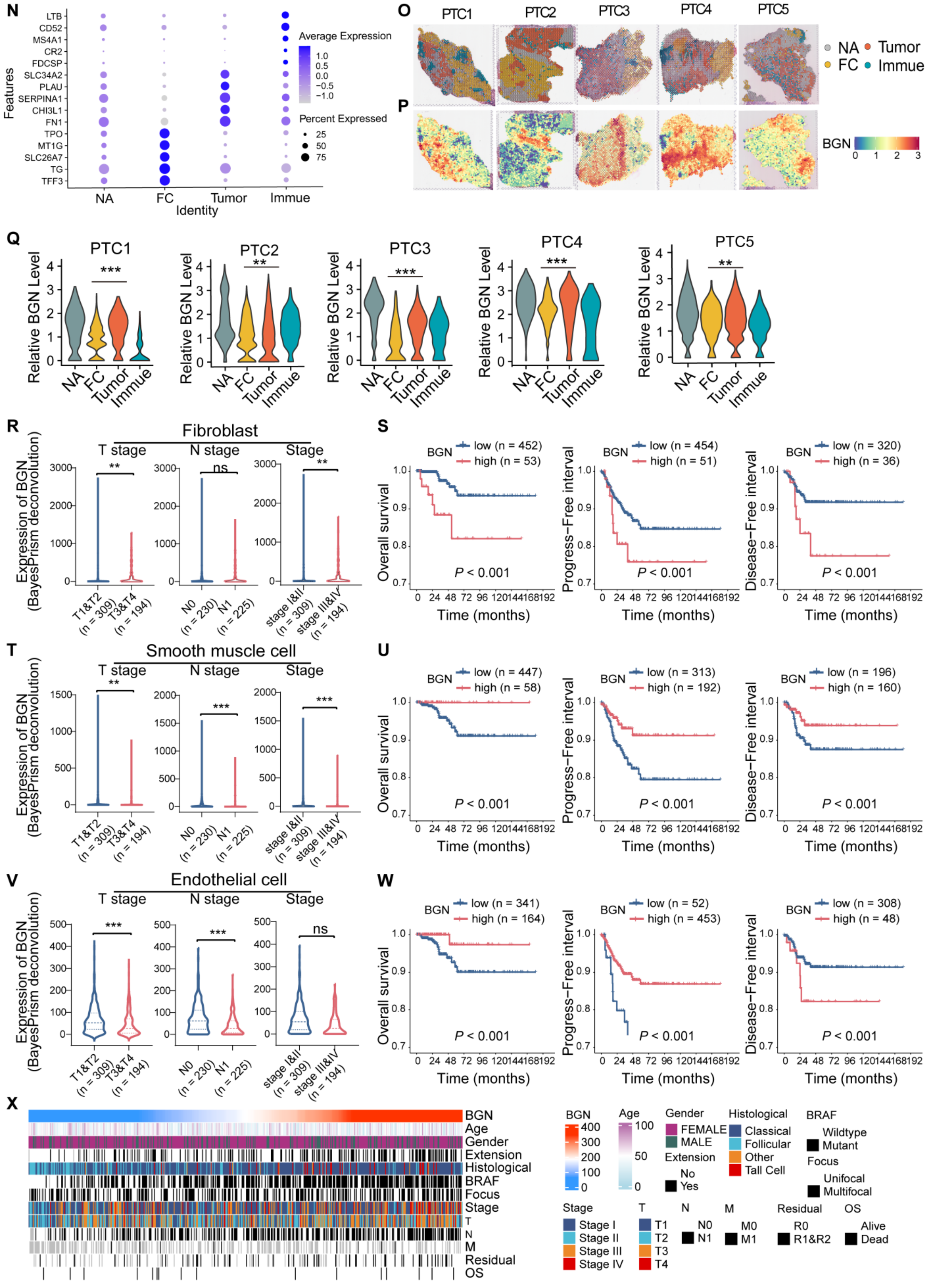

N
O
PTC1
PTC2
PTC3
PTC4
PTC5
NA
Tumor
FC
Immue
P
BGN
Average Expression
Percent Expressed
Features
Identity
Q
Relative BGN Level
R
Fibroblast
T stage
N stage
Stage
Expression of BGN (BayesPrism deconvolution)
S
BGN
Overall survival
Progress-Free interval
Disease-Free interval
Time (months)
T
Smooth muscle cell
U
V
Endothelial cell
W
X
Age
Gender
Extension
Histological
BRAF
Focus
Stage
T
N
M
Residual
OS
FEMALE
MALE
Classical
Follicular
Other
Tall Cell
Wildtype
Mutant
Unifocal
Multifocal
No
Yes
Stage I
Stage II
Stage III
Stage IV
T1
T2
T3
T4
N0
N1
M0
M1
R0
R1&R2
Alive
Dead

Figure S1. (**A, C, E**) t-SNE plots of scRNA-seq datasets GSE250521 (A), GSE193581 (C), and GSE184362 (E) showing sample distribution. (**B, D, F**) Dot plots of cell-type-specific marker genes for each dataset. (**G, J**) t-SNE plots of scRNA-seq datasets GSE193581 (G) and GSE184362 (J) showing cell types distribution. (H, K) BGN expression mapped on t-SNE scatter plots of scRNA-seq datasets GSE193581 (H) and GSE184362 (K). (**I- L**) Violin plots comparing BGN expression between malignant cells and normal thyroid follicular cells in the scRNA-seq datasets GSE193581 (I) and GSE184362 (L). (**M**) Spatial distribution of BGN expression in spatial transcriptomics dataset GSE250521. (**N-O**) Spatial segmentation of normal and tumor regions in five PTC samples using Yan et al.'s framework. (**P-Q**) Distribution (P) and Violin plots (Q) of BGN expression in tumor vs. normal spots across five sections. (**R, T, V**) Violin plots showing differences in relative BGN expression levels in fibroblast (R), smooth muscle cell (T), and endothelial cell (V), as determined by BayesPrism deconvolution analysis, among different clinicopathological subgroups. (**S, U, W**) Kaplan–Meier survival analysis demonstrates that high fibroblast (S), smooth muscle cell (U), and endothelial cell (W) secreted BGN expression, classified by the optimal cutoff from BayesPrism deconvolution analysis, is associated with poorer overall survival (OS), progression-free survival (PFS), and disease-free survival (DFS) in PTC patients. (**X**) Heatmap illustrating the BGN expression levels and their associations with clinicopathological features in the PTC patient cohort. (*$p < 0.05$, **$p < 0.01$, ***$p < 0.001$).

Figure S2

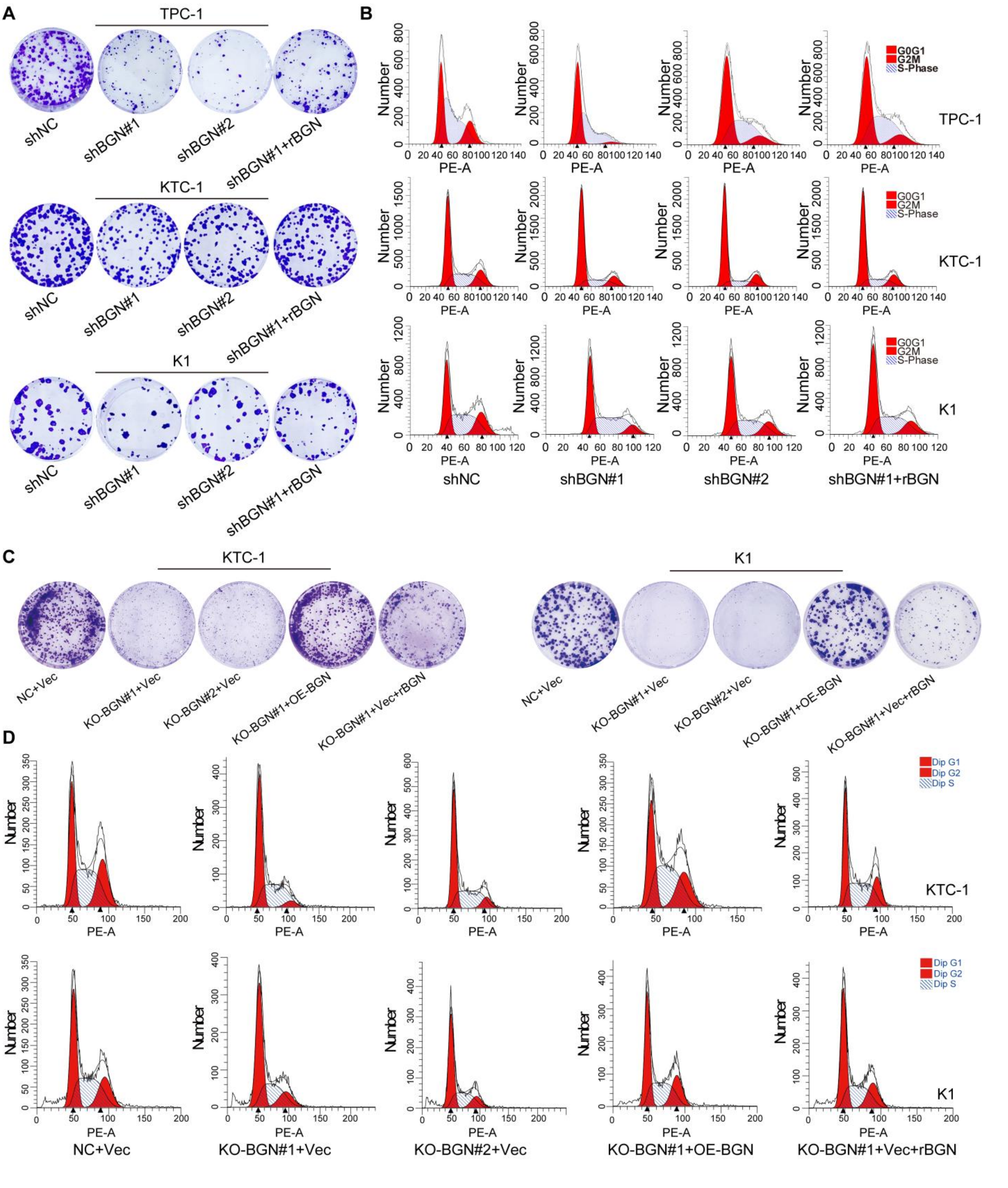

Figure S2. (A) Representative images of colony formation assays showing the impact of BGN knockdewn or rBGN supplement on the proliferation of TPC-1, KTC-1, and K1 cells. (**B**) Representative flow cytometry images illustrating changes in cell cycle distribution in TPC-1, KTC-1, and K1 cells following BGN

knockdown or rBGN supplement. (**C**) Representative images of colony formation assays showing the impact of BGN knockout, BGN overexpression or rBGN supplement on the proliferation of KTC-1 and K1 cells. **(D)** Representative flow cytometry images illustrating changes in cell cycle distribution in KTC-1 and K1 cells following BGN knockout, BGN overexpression or rBGN supplement.

Figure S3

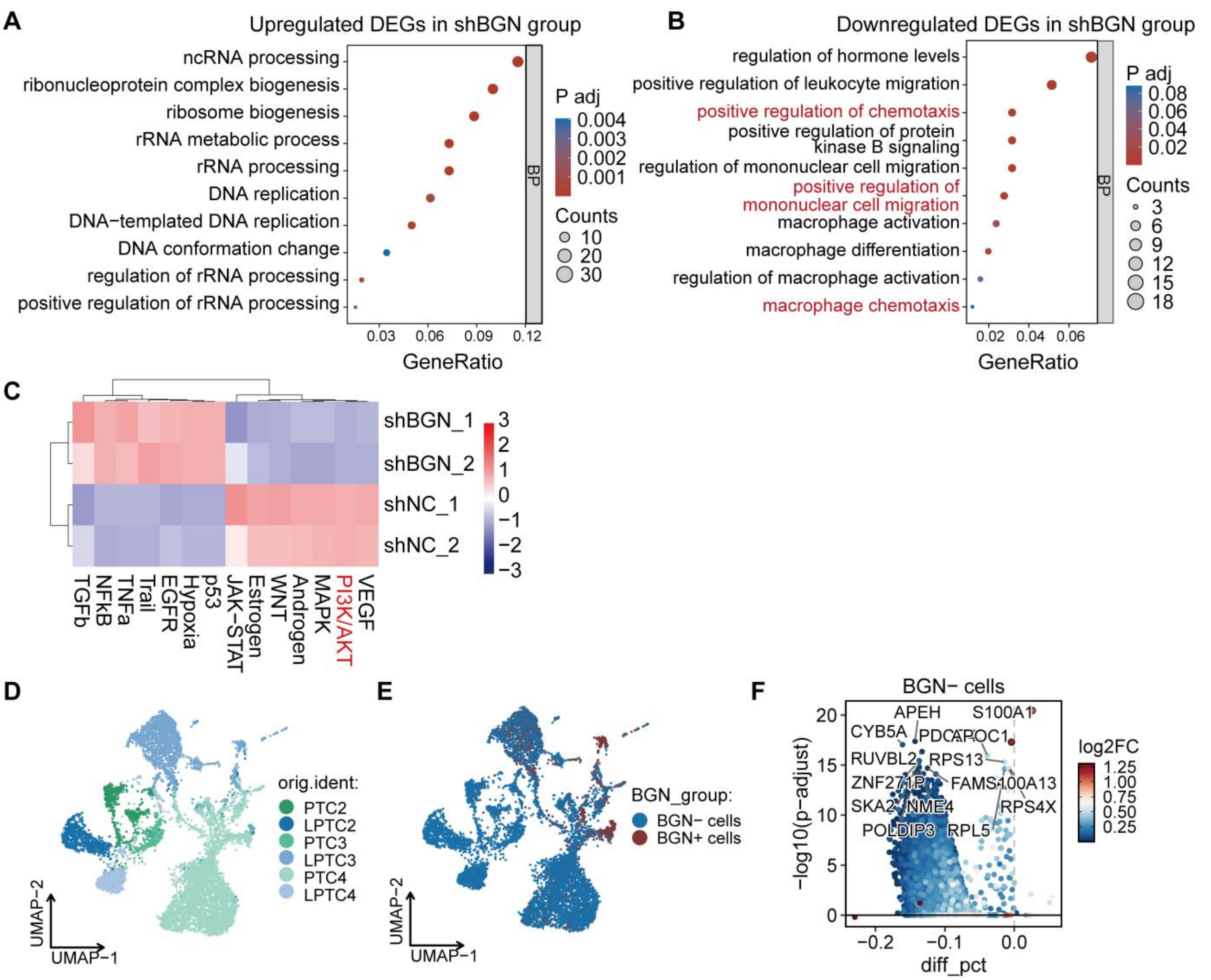

Figure S3. (A-B) GO enrichment analysis revealing significantly enriched biological processes among downregulated (A) and upregulated genes (B) in the shBGN group. **(C)** Heatmap depicting pathway activity in RNA-seq samples analyzed using the "decoupleR" package. **(D)** UMAP scatter plot of scRNA-seq data from PTC tumor samples (GSE250521) showing the samples distribution. **(E)** UMAP scatter plot displaying the distribution of BGN^{+} and BGN^{-} PTC cells across tumor samples. **(F)** Volcano plot showing different expression genes between BGN^{-} and BGN^{+} cells.

Figure S4

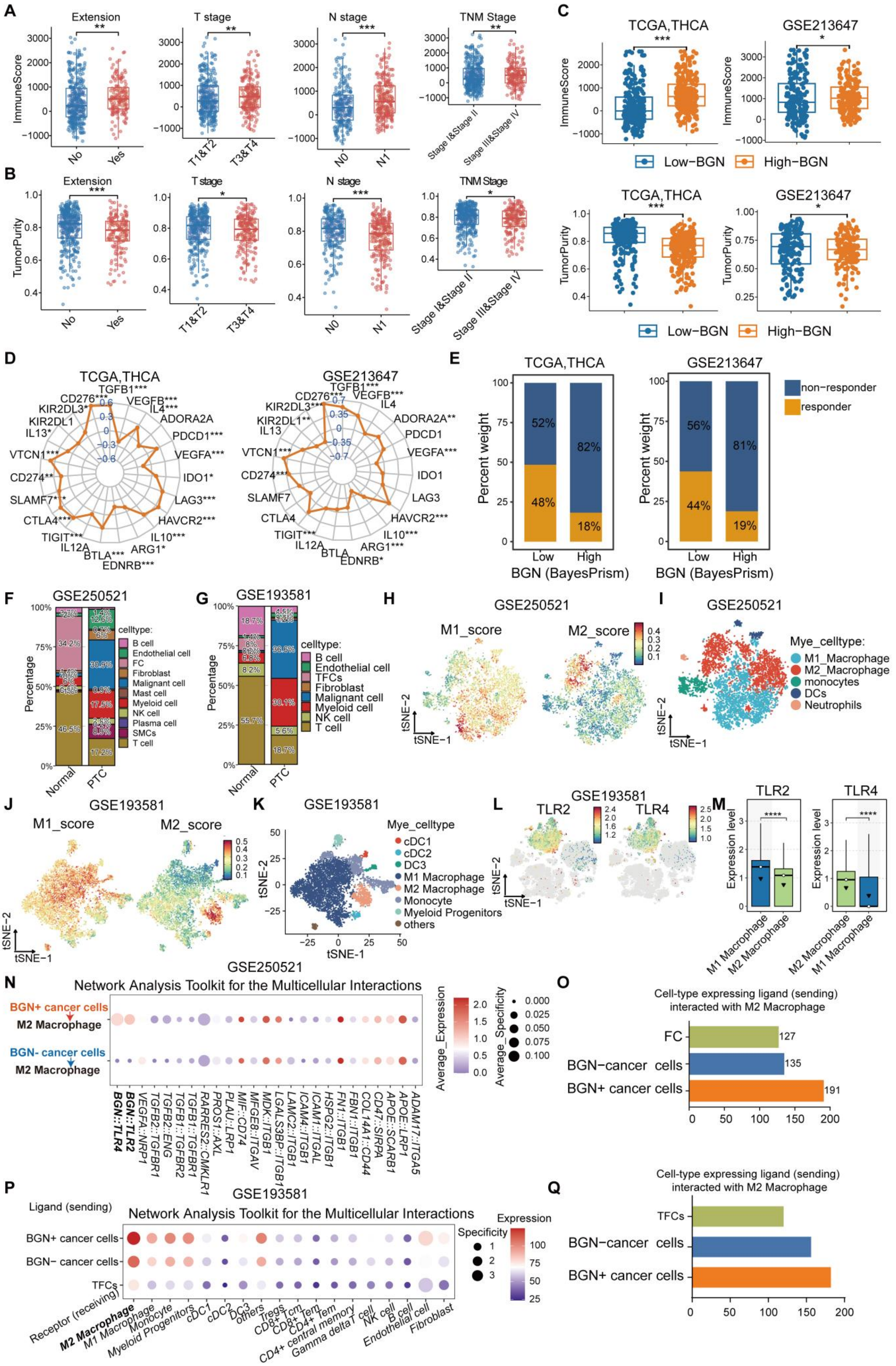

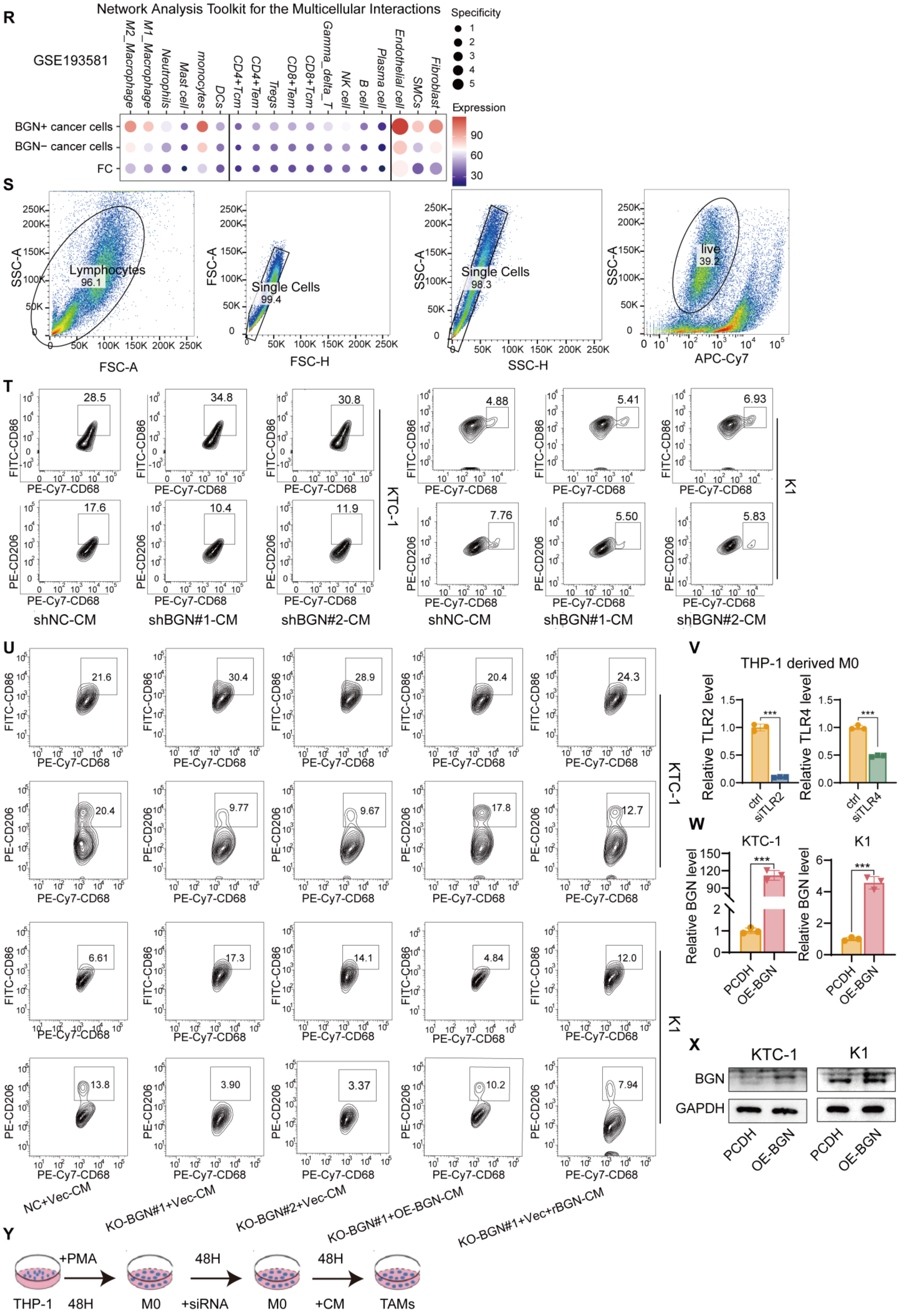

R
Network Analysis Toolkit for the Multicellular Interactions
GSE193581
BGN+ cancer cells
BGN− cancer cells
FC
Specificity
Expression
S
Lymphocytes 96.1
Single Cells 99.4
Single Cells 98.3
live 39.2
FSC-A
FSC-H
SSC-H
APC-Cy7
T
KTC-1
K1
shNC-CM
shBGN#1-CM
shBGN#2-CM
U
KTC-1
K1
NC+Vec-CM
KO-BGN#1+Vec-CM
KO-BGN#2+Vec-CM
KO-BGN#1+OE-BGN-CM
KO-BGN#1+Vec+rBGN-CM
V
THP-1 derived M0
Relative TLR2 level
Relative TLR4 level
ctrl
siTLR2
siTLR4
W
KTC-1
K1
Relative BGN level
PCDH
OE-BGN
X
KTC-1
K1
BGN
GAPDH
PCDH
OE-BGN
Y
THP-1
+PMA
48H
M0
+siRNA
48H
M0
+CM
48H
TAMs

Figure S4. (A-B) Correlation between immune scores (A) or tumor purity (B) and advanced clinical features (extrathyroid invasion, T3/4 stage, N1 stage, TNM III/IV) in the TCGA-THCA dataset. **(C)** Immune score and tumor purity analysis between PTC patients with low and high BGN expression groups based on TCGA and GSE213647 datasets. BayesPrism was applied to obtain tumor cell-specific expression profiles. **(D)** Correlation analysis between BGN expression and immunosuppressive genes. BayesPrism was applied to obtain tumor cell-specific expression profiles. **(E)** Response rate of PTC patients with high BGN expression to immune checkpoint blockade (ICB) therapy. BayesPrism was applied to obtain tumor cell-specific expression profiles. **(F-G)** The cell-type proportions between PTC and normal tissues in the GSE250521 (F) and GSE193581 (G) scRNA-seq datasets**.** **(H)** t-SNE plot showing the expression of M1 and M2 macrophage scores in myeloid cells from the GSE250521 dataset. **(I)** t-SNE plot illustrating the distribution of myeloid cell subpopulations in the GSE250521 dataset. **(J)** t-SNE plot showing M1 and M2 macrophage scores in myeloid cells from the GSE193581 dataset. **(K)** t-SNE plot displaying the distribution of myeloid cell subpopulations in the GSE193581 dataset. (**L**) t-SNE scatter plot showing the distribution of TLR2 (left) and TLR4 (right) in the GSE193581 single-cell RNA-seq dataset. (**M**) Box plot showing the expression levels of TLR2 and TLR4 in M1 and M2 macrophages from the GSE193581 dataset. **(N)** Bubble plot showing the interaction specificity and strength between BGN+ and BGN- PTC tumor cells, and normal thyroid cells, with microenvironmental cells in the GSE250521 dataset by NATMI. **(O)** Bar graph showing the number of interactions between BGN+ PTC tumor cells, BGN- tumor cells, and normal thyroid cells with M2 macrophages in the GSE250521 dataset. **(P)** Bubble plot depicting the communication strength and specificity between BGN+ and BGN- PTC tumor cells, and normal thyroid cells with microenvironmental cells in the GSE193581 dataset by NATMI. **(Q)** Bar graph showing the number of interactions between BGN+ PTC tumor cells, BGN- tumor cells, and normal thyroid cells with M2 macrophages in the GSE193581 dataset. (**R**) Bubble plot displaying the ligand-receptor interactions between BGN+ tumor cells and M2 macrophages in the GSE193581 dataset by NATMI. (**S**) Gating strategy for macrophage flow

cytometry analysis. **(T)** Representative flow cytometry results of macrophages co-cultured with CM from control and BGN-knockdown groups. **(U)** Representative flow cytometry plots showing M1 and M2 macrophage populations after incubation with CM from the BGN knockout group compared to the control group, and with CM from the BGN overexpression and rBGN supplementation groups compared to the BGN knockout group. **(V)** qRT-PCR validation of TLR2 or TLR4 knockdown efficiency in THP-1-derived macrophages using siRNA. **(W)** qRT-PCR analysis of BGN mRNA levels in KTC-1 and K1 cells with BGN overexpression (OE-BGN) or control (PCDH). **(X)** Western blot analysis of BGN protein levels in KTC-1 and K1 cells with BGN overexpression (OE-BGN) or control (PCDH). **(Y)** Schematic diagram illustrating the experimental design of THP-1-derived macrophages treated with siRNA targeting TLR2 or TLR4, followed by co-culture with CM from BGN-overexpression and control groups. (*$p < 0.05$, **$p < 0.01$, ***$p < 0.001$).

Figure S5

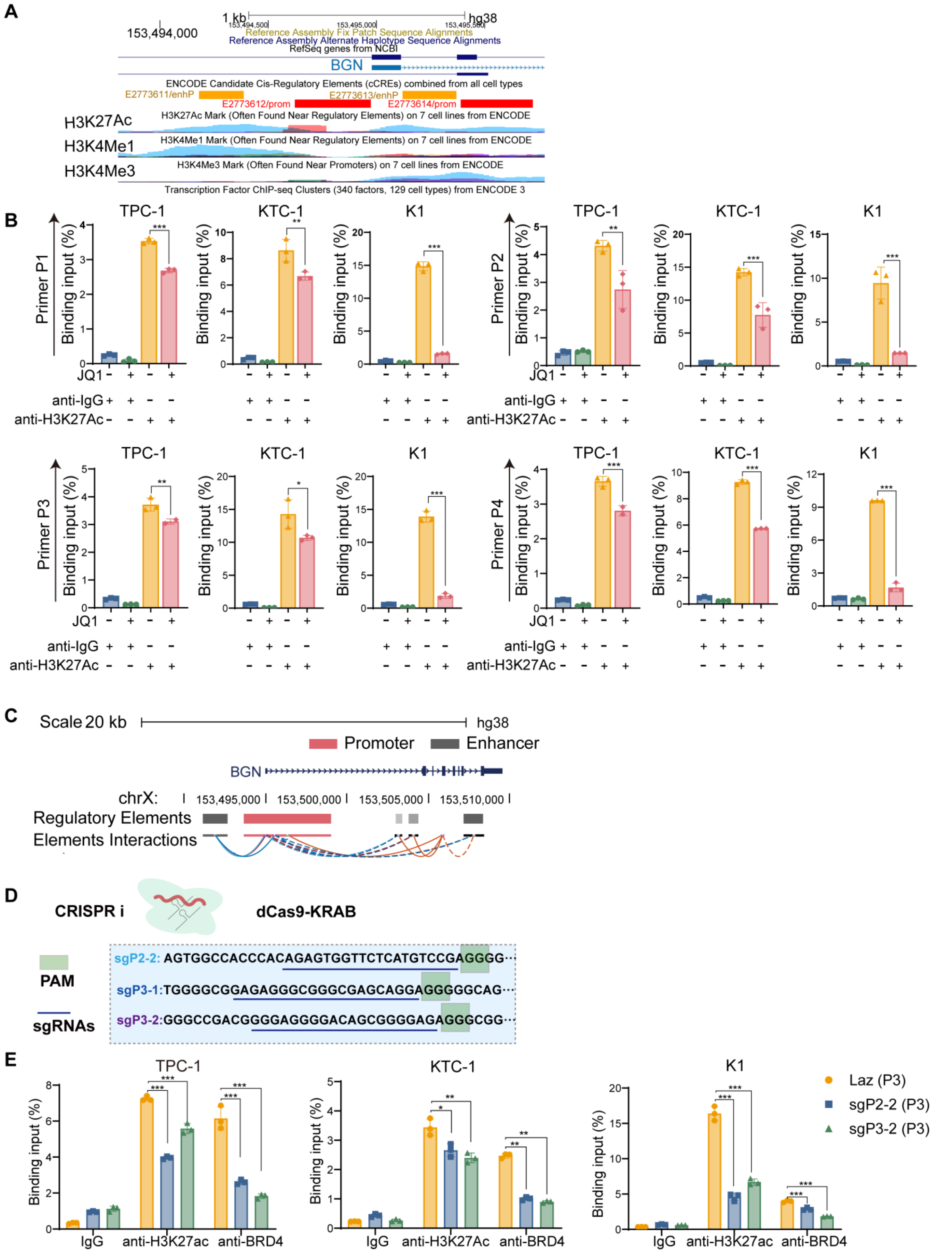

Figure S5. (A) H3K27ac, H3K4me1, and H3K4me3 modification profiles at the BGN enhancer and promoter regions based on ENCODE data. **(B)** ChIP-qPCR analysis of H3K27ac modification levels at the

BGN enhancer region after JQ1 treatment. **(C)** GeneHancer database showing that the BGN enhancer regulates the expression of the downstream BGN gene. (**D**) Schematic diagram of CRISPRi targeting the BGN enhancer. **(E)** ChIP-qPCR analysis of H3K27ac and BRD4 levels at the P3 primer region following CRISPRi-mediated knockdown of the BGN enhancer. (*$p < 0.05$, **$p < 0.01$, ***$p < 0.001$).

Figure S6

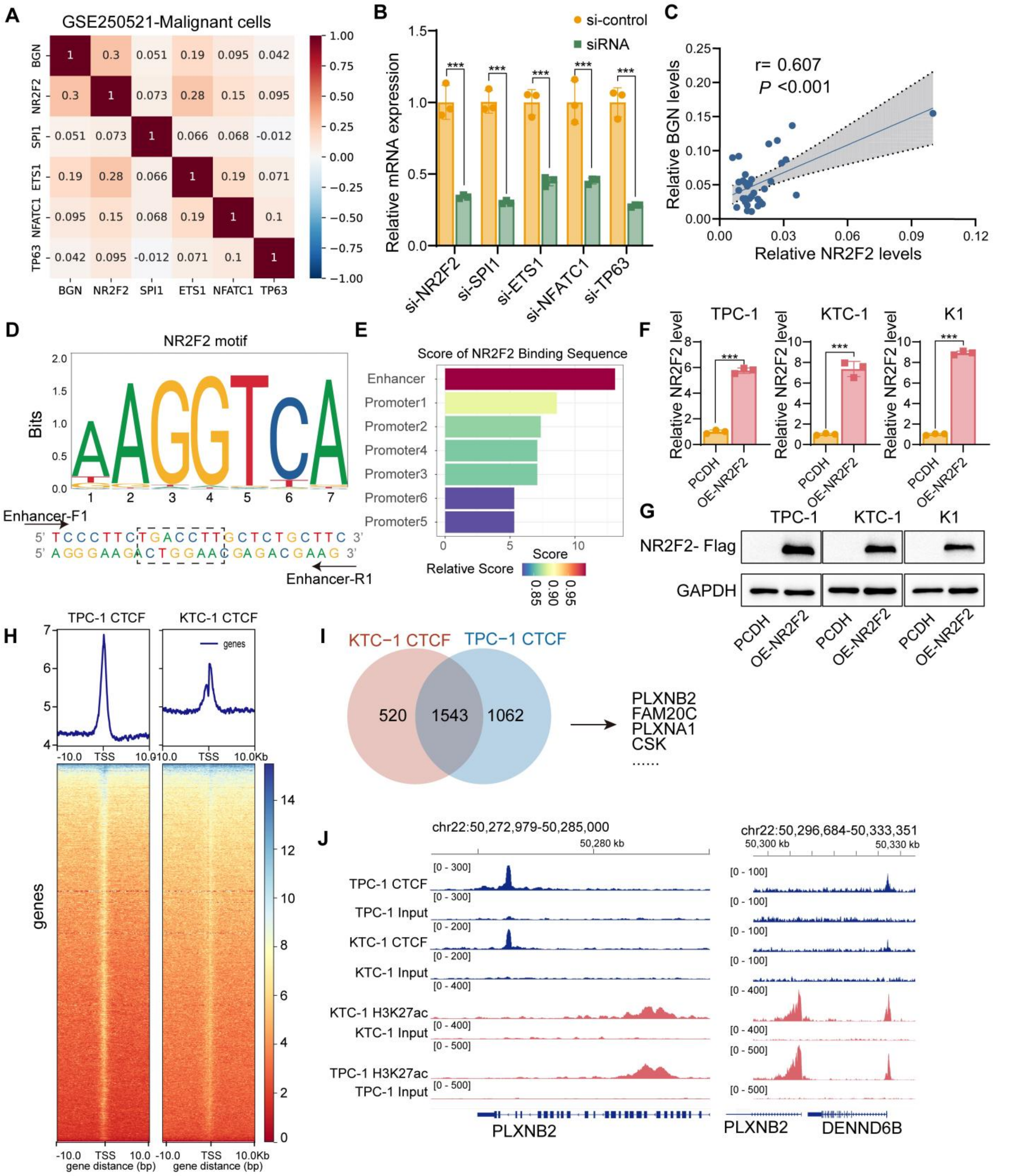

Figure S6. (A) Correlation analysis of five transcription factors and BGN expression in malignant PTC cell subpopulations using scRNA-seq data. **(B)** qRT-PCR validation of knockdown efficiency for NR2F2, SPI1, ETS1, NFATC1, and TP63 using siRNA. **(C)** Correlation analysis of NR2F2 and BGN mRNA levels in PTC

tissues. **(D)** JASPAR database showing the NR2F2 motif and predicted binding sites at the BGN enhancer. **(E)** JASPAR database predicting binding scores of NR2F2 at the BGN enhancer and promoter. (**F-G)** qRT-PCR (F) and western blot (G) analysis of NR2F2-Flag protein and BGN protein expression in PTC cell lines overexpressing NR2F2. (**H**) The peak plots and Heatmap showing CTCF ChIP-seq enrichment within ± 10 kb of transcription start sites (TSS) across the genome in TPC-1 and KTC-1 cells. (**I**) The Venn diagram showing the intersection of genes annotated from CTCF peak regions in KTC-1 and TPC-1 cells. (**J**) ChIP-seq tracks for CTCF binding at the PLXNB2 gene promoter and enhancer. (*$p < 0.05$, **$p < 0.01$, ***$p < 0.001$).

Figure S7

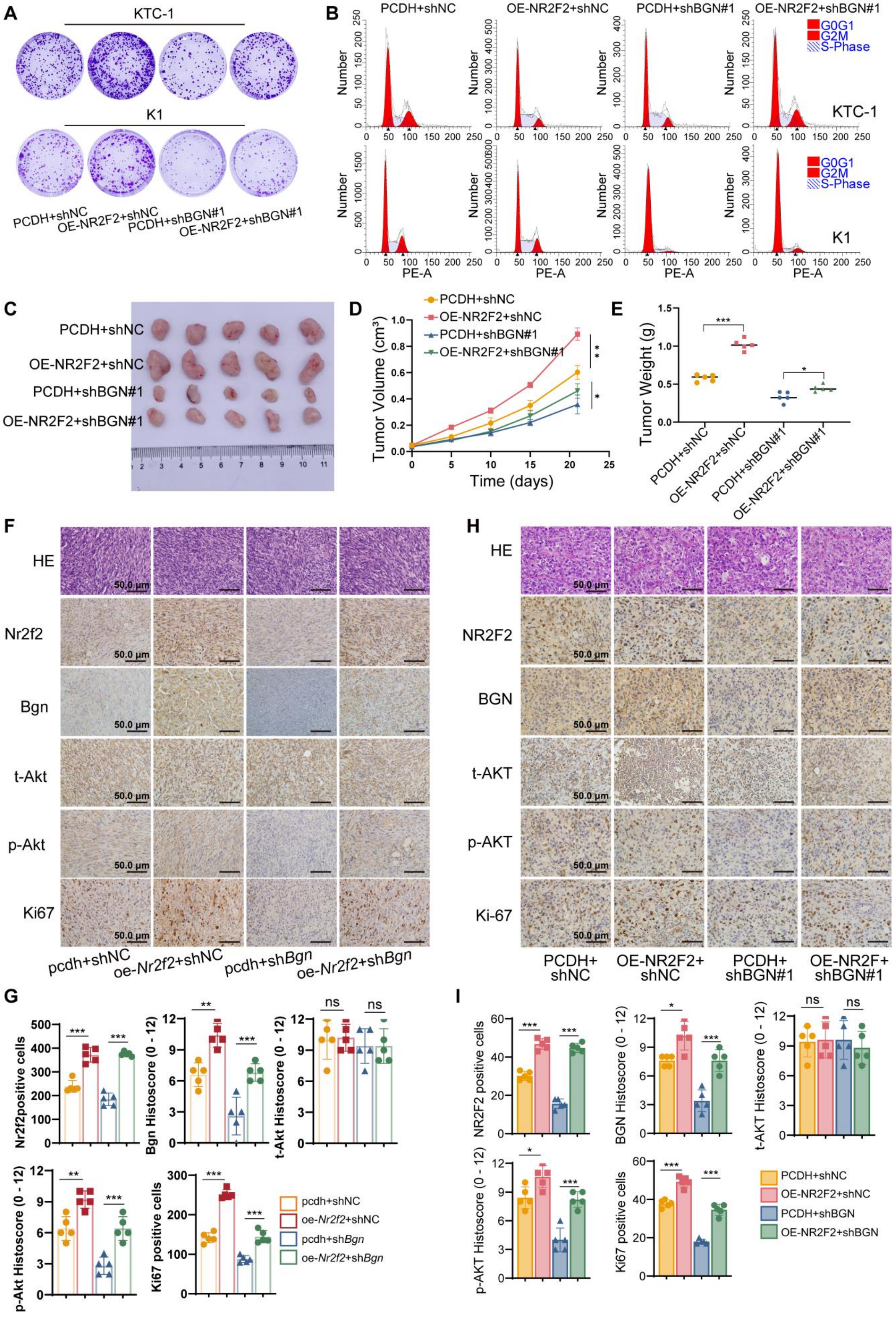

A
KTC-1
K1
PCDH+shNC
OE-NR2F2+shNC
PCDH+shBGN#1
OE-NR2F2+shBGN#1
B
PCDH+shNC
OE-NR2F2+shNC
PCDH+shBGN#1
OE-NR2F2+shBGN#1
G0G1
G2M
S-Phase
KTC-1
K1
Number
PE-A
C
PCDH+shNC
OE-NR2F2+shNC
PCDH+shBGN#1
OE-NR2F2+shBGN#1
D
Tumor Volume (cm³)
Time (days)
E
Tumor Weight (g)
F
HE
Nr2f2
Bgn
t-Akt
p-Akt
Ki67
50.0 µm
pcdh+shNC
oe-Nr2f2+shNC
pcdh+shBgn
oe-Nr2f2+shBgn
H
HE
NR2F2
BGN
t-AKT
p-AKT
Ki-67
PCDH+ shNC
OE-NR2F2+ shNC
PCDH+ shBGN#1
OE-NR2F+ shBGN#1
G
Nr2f2positive cells
Bgn Histoscore (0 - 12)
t-Akt Histoscore (0 - 12)
p-Akt Histoscore (0 - 12)
Ki67 positive cells
I
NR2F2 positive cells
BGN Histoscore (0 - 12)
t-AKT Histoscore (0 - 12)
p-AKT Histoscore (0 - 12)
Ki67 positive cells
PCDH+shNC
OE-NR2F2+shNC
PCDH+shBGN
OE-NR2F2+shBGN

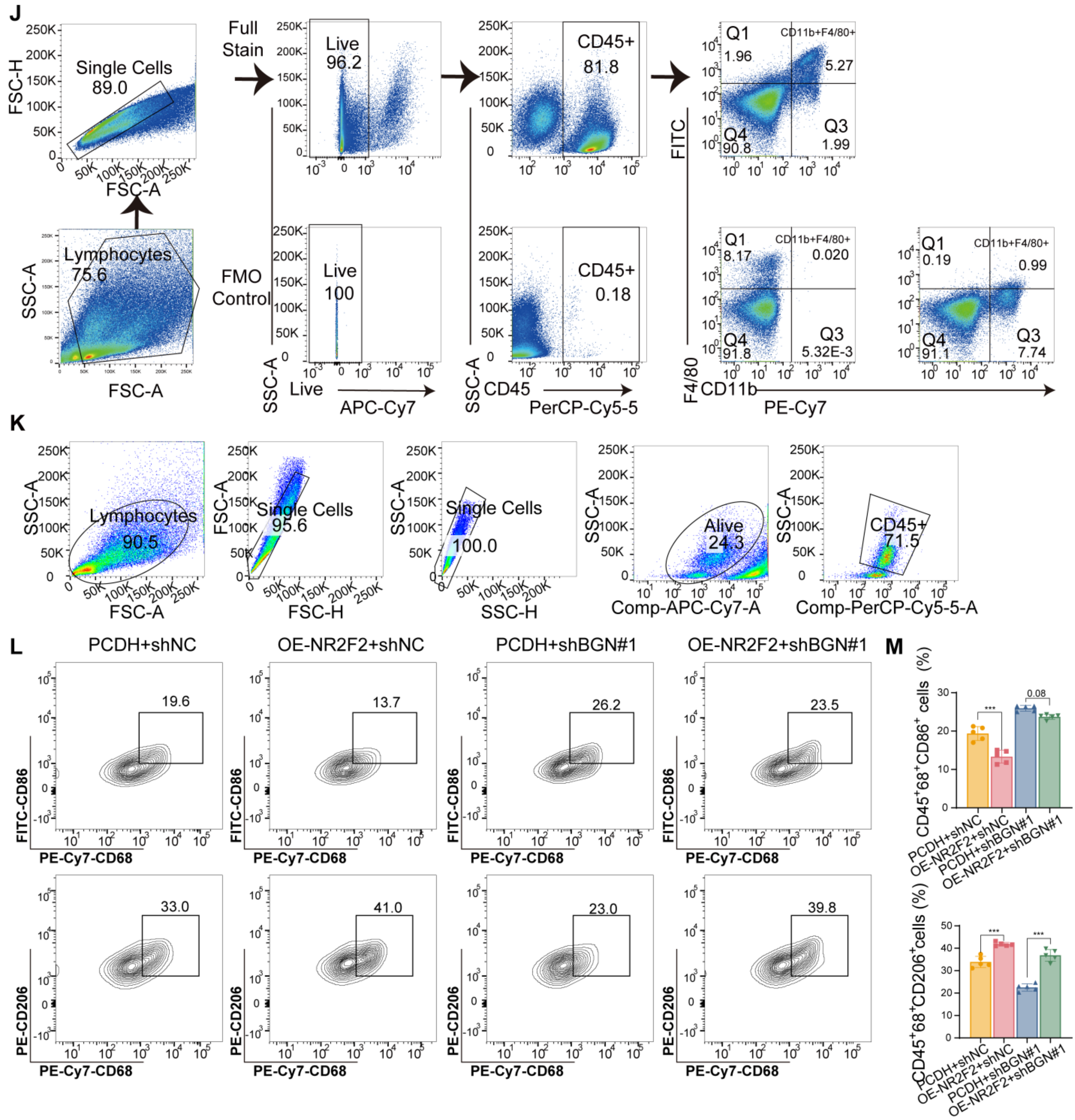

Figure S7. **(A)** Representative images of colony formation assays showing the effects of NR2F2 overexpression and BGN knockdown in KTC-1 and K1 cells. (**B**) Representative flow cytometry images illustrating changes in cell cycle distribution in KTC-1 and K1 cells following the NR2F2 overexpression and BGN knockdown. **(C)** Schematic representation of the nude mouse xenograft model and tumor tissue isolation. **(D)** Tumor growth curves illustrating the effect of the NR2F2-BGN axis on subcutaneous tumor growth in nude mice. **(E)** Tumor weight comparison demonstrating the impact of the NR2F2-BGN axis on

subcutaneous tumor burden. **(F)** Representative immunohistochemical staining images of Nr2f2, Bgn, total Akt, p-Akt, and Ki67 in subcutaneous tumor of C57BL/6J mice treated with PBS liposomes. **(G)** Comparative analysis of immunohistochemical staining scores for Nr2f2, Bgn, total Akt, p-Akt, and Ki67 in subcutaneous tumor of C57BL/6J mice treated with PBS liposomes. **(H)** Representative immunohistochemical staining images of NR2F2, BGN, total AKT, p-AKT, and Ki67 in subcutaneous tumor of nude mice co-injection with THP-1 cells. **(I)** Comparative analysis of immunohistochemical staining scores for NR2F2, BGN, total AKT, p-AKT, and Ki67 in subcutaneous tumor of nude mice co-injection with THP-1 cells. **(J)** Flow cytometry gating strategy for analyzing the proportions of macrophages in subcutaneous tumor of C57BL/6J mice treated with PBS liposomes and Clodronate liposome. **(K)** Flow cytometry gating strategy for analyzing the proportions of macrophages in subcutaneous tumor of nude mice co-injection with THP-1 cells. **(L)** Representative flow cytometry plots showing the proportion of M1 ($CD45^+CD68^+CD86^+$) and M2 macrophages in different groups. **(M)** Quantification of M1 ($CD45^+CD68^+CD86^+$) and M2 ($CD45^+CD68^+CD206^+$) macrophage populations in nude mice subcutaneous tumor tissues. (*$p < 0.05$, **$p < 0.01$, ***$p < 0.001$).

Figure S8

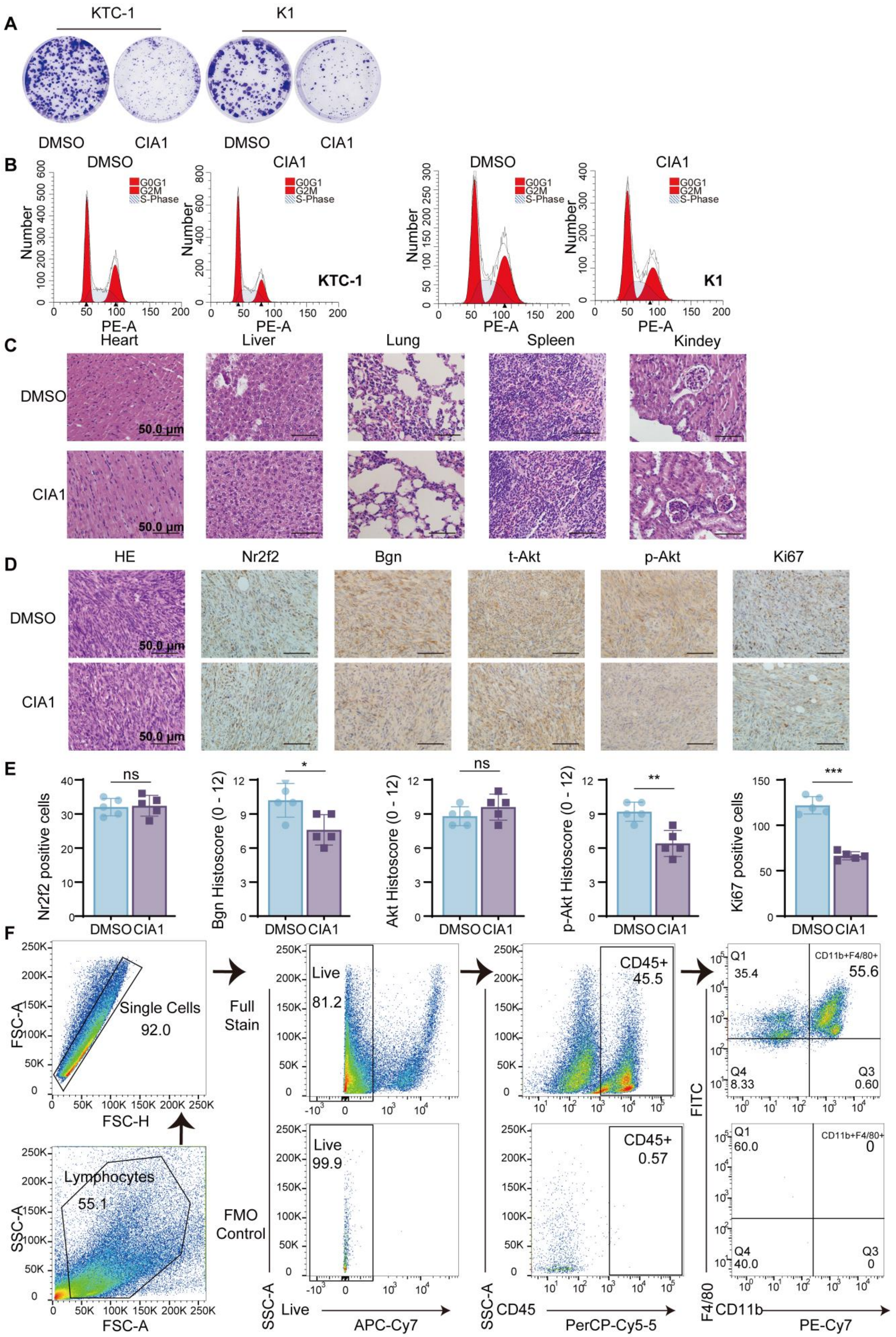
A
KTC-1
K1
DMSO
CIA1
DMSO
CIA1
B
DMSO
CIA1
DMSO
CIA1
G0G1
G2M
S-Phase
Number
PE-A
KTC-1
K1
C
Heart
Liver
Lung
Spleen
Kindey
DMSO
CIA1
50.0 μm
D
HE
Nr2f2
Bgn
t-Akt
p-Akt
Ki67
DMSO
CIA1
50.0 μm
E
ns
*
ns
**
***
Nr2f2 positive cells
Bgn Histoscore (0 - 12)
Akt Histoscore (0 - 12)
p-Akt Histoscore (0 - 12)
Ki67 positive cells
DMSO
CIA1
F
Single Cells
92.0
FSC-A
FSC-H
Lymphocytes
55.1
SSC-A
FSC-A
Full Stain
FMO Control
Live
81.2
Live
99.9
SSC-A
Live
APC-Cy7
CD45+
45.5
CD45+
0.57
SSC-A
CD45
PerCP-Cy5-5
Q1
35.4
CD11b+F4/80+
55.6
Q4
8.33
Q3
0.60
Q1
60.0
CD11b+F4/80+
0
Q4
40.0
Q3
0
FITC
F4/80
CD11b
PE-Cy7

Figure S8. (**A**) Representative images of colony formation assays showing the impact of 5 μM CIA1 treatment on KTC-1 and K1 cells proliferation. (**B**) Representative flow cytometry images illustrating changes in cell cycle distribution in KTC-1 and K1 cells following 5 μM CIA1 treatment. (**C**) HE staining to assess tissue morphology in the heart, liver, and kidneys of mice treated with CIA1 (scale bar: 50 μM). (**D**) Representative IHC staining of tumor tissues from DMSO vs CIA1-treated mice showing Nr2f2, Bgn, Akt, p-Akt, and Ki67 expression (scale bar: 50 μM). (**E**) IHC analysis of positive signal intensity for various tumor markers in DMSO vs CIA1-treated tumors. (**F**) Gating strategy for macrophage flow cytometry analysis in subcutaneous murine tumor tissues. (*$p < 0.05$, **$p < 0.01$, ***$p < 0.001$).

Table S1. The samples information for scRNA-seq and spatial transcriptomic datasets in this study.

Sample name	Source	ID	Tissue type	Data type
N2	GSE250521	GSM7980876	Para-tumor	scRNA-seq
N3	GSE250521	GSM7980877	Para-tumor	scRNA-seq
N4	GSE250521	GSM7980878	Para-tumor	scRNA-seq
PTC2	GSE250521	GSM7980879	PTC	scRNA-seq
PTC3	GSE250521	GSM7980880	PTC	scRNA-seq
PTC4	GSE250521	GSM7980881	PTC	scRNA-seq
LPTC2	GSE250521	GSM7980882	LPTC	scRNA-seq
LPTC3	GSE250521	GSM7980883	LPTC	scRNA-seq
LPTC4	GSE250521	GSM7980884	LPTC	scRNA-seq
NORM03	GSE193581	GSM5814577	Para-tumor	scRNA-seq
NORM07	GSE193581	GSM5814582	Para-tumor	scRNA-seq
NORM18	GSE193581	GSM5814593	Para-tumor	scRNA-seq
NORM19	GSE193581	GSM5814594	Para-tumor	scRNA-seq
NORM20	GSE193581	GSM5814595	Para-tumor	scRNA-seq
NORM21	GSE193581	GSM5814596	Para-tumor	scRNA-seq
PTC01	GSE193581	GSM5814574	PTC	scRNA-seq
PTC02	GSE193581	GSM5814575	PTC	scRNA-seq
PTC03	GSE193581	GSM5814576	PTC	scRNA-seq
PTC04	GSE193581	GSM5814578	PTC	scRNA-seq
PTC05	GSE193581	GSM5814579	PTC	scRNA-seq
PTC06	GSE193581	GSM5814580	PTC	scRNA-seq
PTC07	GSE193581	GSM5814581	PTC	scRNA-seq
PTC1_P	GSE184362	GSM5585103	Para-tumor	scRNA-seq
PTC2_P	GSE184362	GSM5585105	Para-tumor	scRNA-seq
PTC3_P	GSE184362	GSM5585108	Para-tumor	scRNA-seq
PTC5_P	GSE184362	GSM5585113	Para-tumor	scRNA-seq
PTC8_P	GSE184362	GSM5585118	Para-tumor	scRNA-seq
PTC9_P	GSE184362	GSM5585120	Para-tumor	scRNA-seq
PTC1_T	GSE184362	GSM5585102	PTC	scRNA-seq
PTC2_T	GSE184362	GSM5585104	PTC	scRNA-seq
PTC3_T	GSE184362	GSM5585107	PTC	scRNA-seq
PTC5_T	GSE184362	GSM5585112	PTC	scRNA-seq
PTC8_T	GSE184362	GSM5585117	PTC	scRNA-seq
PTC9_T	GSE184362	GSM5585119	PTC	scRNA-seq
PTC10_T	GSE184362	GSM5585121	PTC	scRNA-seq
N1	GSE250521	GSM7980860	Para-tumor	spRNA-seq

Sample name	Source	ID	Tissue type	Data type
N2	GSE250521	GSM7980861	Para-tumor	spRNA-seq
N3	GSE250521	GSM7980862	Para-tumor	spRNA-seq
N4	GSE250521	GSM7980863	Para-tumor	spRNA-seq
PTC1	GSE250521	GSM7980864	PTC	spRNA-seq
PTC2	GSE250521	GSM7980865	PTC	spRNA-seq
PTC3	GSE250521	GSM7980866	PTC	spRNA-seq
PTC4	GSE250521	GSM7980867	PTC	spRNA-seq
LPTC1	GSE250521	GSM7980868	LPTC	spRNA-seq
LPTC2	GSE250522	GSM7980869	LPTC	spRNA-seq
LPTC3	GSE250521	GSM7980870	LPTC	spRNA-seq
LPTC4	GSE250521	GSM7980871	LPTC	spRNA-seq
PTC1	HRA003537	HRS493177	PTC	spRNA-seq
PTC2	HRA003537	HRS493178	PTC	spRNA-seq
PTC3	HRA003537	HRS493179	PTC	spRNA-seq
PTC4	HRA003537	HRS493180	PTC	spRNA-seq
PTC5	HRA003537	HRS493181	PTC	spRNA-seq

Table S2. Sequences of shRNA, sgRNA and siRNA used in the study.

**shRNA / siRNA**	**5'-3'**
shBGN#1	GAAGCTCTACATCTCCAAGAA
shBGN#2	GAACATGAACTGCATCGAGAT
shBGN#3	GCCATTCATGATGAACGATGA
KO-BGN#1-sgRNA	caccGAGAGACACGAGGCGCCACAg
KO-BGN#2-sgRNA	caccGGCCATTCATGATGAACGATg
KO-BGN#3-sgRNA	caccGCCTTGCGGATGCGGTTGTCg
shBgn	CTCCCTGGTAGAACTACGAAT
siTLR2 sense	GGAAGAUAAUGAACACCAATT
siTLR2 anti-sense	UUGGUGUUCAUUAUCUUCCTT
siTLR4 sense	CCAGGUGCAUUUAAAGAAATT
siTLR4 anti-sense	UUUCUUUAAAUGCACCUGGTT
si-NR2F2 sense	GCGAGCTGTTTGTGTTGAATT
si-NR2F2 anti-sense	TTCAACACAAACAGCTCGCTT
si-SPI1 sense	CCUAUGACACGGAUCUAUACC
si-SPI1 anti-sense	UAUAGAUCCGUGUCAUAGGGC
si-ETS1 sense	CCGUGCUGACCUCAAUAAGTT
si-ETS1 anti-sense	CUUAUUGAGGUCAGCACGGTT
si-TP63 sense	GUGUGCUGGUACCUUAUGATT
si-TP63 anti-sense	UCAUAAGGUACCAGCACACTT
si-NFATC1 sense	GGACUCCAAGGUCAUUUUC
si-NFATC1 anti-sense	GAAAAUGACCUUGGAGUCC

Table S3. Primer sequences for quantitative real-time PCR.

Primers	5'-3'
BGN-F	GAGACCCTGAATGAACTCCACC
BGN-R	CTCCCGTTCTCGATCATCCTG
ACTB-F	CTCCTTAATGTCACGCACGAT
ACTB-R	CATGTACGTTGCTATCCAGGC
ATF1-F	TCCGACAGCATAGGCTCCTCAC
ATF1-R	CTGTGCCTGGACTTGCCAACTG
MAPK9-F	CGCCCGAAGTCATCCTGGGTAT
MAPK9-R	AGGATACGGTCAGTGCCTTGGA
DDIT4-F	CTTGTGTGCCAACCTGATGC
DDIT4-R	GGAGAGTTGGCGGAGCTAAA
CDKN1A-F	CGATGGAACTTCGACTTTGTCA
CDKN1A-R	GCACAAGGGTACAAGACAGTG
PPP2R5B-F	ACATCCGCAAACAGTGCAAC
PPP2R5B-R	CCCCCAGAAACATCACCTCC
EGFR-F	AGACATGGACGACGTGGTGGAT
EGFR-R	GGAGGTTGAGGAGCAGGACTGT
CD86-F	CCATCAGCTTGTCTGTTTCATTCC
CD86-R	GCTGTAATCCAAGGAATGTGGTC
CD11c-F	GATGCTCAGAGATACTTCACGGC
CD11c-R	CCACACCATCACTTCTGCGTTC
Arg1-F	TCATCTGGGTGGATGCTCACAC
Arg1-R	GAGAATCCTGGCACATCGGGAA
CD163-F	CCAGAAGGAACTTGTAGCCACAG
CD163-R	CAGGCACCAAGCGTTTTGAGCT
TLR2-F	ATCCTCCAATCAGGCTTCTCT
TLR2-R	GGACAGGTCAAGGCTTTTTACA
TLR4-F	AGTTGATCTACCAAGCCTTGAGT
TLR4-R	GCTGGTTGTCCCAAAATCACTTT
P1-F	CACTGGAGGAACTGGACTCATTTGG
P1-R	GCCCTGTGCTGGTCACTTACG
P2-F	TCCTCTGCCTGGCGAGATGC
P2-R	ACAGCAAAGGAACGGACACACTC
P3-F	GCCGCCTCTGTCTCCCTCTC
P3-R	ACGTCTATCTGTCCGGTGTGTCC
P4-F	GCTCTGCTTCATCCACCTCTTGTC
P4-R	AGTCACCCAGCCACGCTCTAG
Enhancer-F	CTGTCAGGCTTCTGCCTCGT
Enhancer-R	CGAGCGGGAGGCAGAGA
Promoter 1-F	CTGTGGGCTCAGGAGGGC
Promoter 1-R	CACCTGTGGACAGAGGGGC
Promoter 2-F	GGCAGGCATGTTTATTCCCACT
Promoter 2-R	CCCTACAGGAAGAGGCTGGAG
Control-F	GGGCGTGCAGTTATGGCTTA
Control-R	CACCTTGCAGCTCTTACCTGA
NR2F2-F	CTCACCTGGAGCGAGCTGTT
NR2F2-R	AGGGAAGGGAGGCGAAGCAA
SPI1-F	TGGTGGGTGGACAAGGACAAGG
SPI1-R	TTCGCCGCTGAACTGGTAGGT
ETS1-F	GATAGTTGTGATCGCCTCACC
ETS1-R	GTCCTCTGAGTCGAAGCTGTC
NFATC1-F	TGGAGAAGCAGAGCACGGACAG
NFATC1-R	TGGCGGGAAGGTAGGTGAAACG
TP63-F	GGAAAACAATGCCCAGACTC
TP63-R	GGACTGGTGGACGAGGAG

Table S4. The detailed sequence of 3C primers.

3C primers	5'-3'
**3C-2-252bp-** enhancr	TGAGTAGCTGCTTTCGGTCCG
3C-1-248bp- enhancr	CATGCCCCCAGGTAGAGCT
3C-1-111bp- enhancr	CCGCCTCTCTCTAGCCACC
3C-3-263bp- enhancr	CCAGGCACCTTTCCCTCAC
**3C-1-193bp-** promoter	GGGCGGAGAAAGAGGGGG
3C-2-168bp- promoter	AGTCTGCAGGCCCCCA
3C-3-223bp- promoter	GCCGAAAGGACACATGGCG
3C-4-302bp- promoter	GGGCGGAGGAGGTGAAGG

Table S5. The detailed sequence of sgRNAs used for CRISPR/Cas9-mediated interference of BGN enhancer.

**sgRNA**	**序列**
sgP2-2-F	caccgAGAGTGGTTCTCATGTCCGA
sgP2-2-R	aaacTCGGACATGAGAACCACTCTc
sgP3-1-F	caccgGAGAGGGCGGGCGAGCAGGA
sgP3-1-R	aaacTCCTGCTCGCCCGCCCTCTCc
sgP3-2-F	caccgGGGAGGGGACAGCGGGGAGA
sgP3-2-R	aaacTCTCCCCGCTGTCCCCTCCCc

Table S8. Predicted binding interactions of NR2F2 at the BGN enhancer and promoter according to the JASPAR database.

Name	Score	Relative score	Sequence ID	Start	End	Strand	Predicted sequence
MA1111.2.NR2F2	13.0861810	0.999999999772634	BGNenhancer	1647	1653	-	AAGGTCA
MA1111.2.NR2F2	8.6034810	0.894409179055670	BGNpromoter	1108	1114	-	AGGGTCA
MA1111.2.NR2F2	7.3657150	0.865253363691999	BGNpromoter	868	874	+	AAGGTGA
MA1111.2.NR2F2	7.0944977	0.858864788492441	BGNpromoter	238	244	+	AAGGCCA
MA1111.2.NR2F2	7.0944977	0.858864788492441	BGNpromoter	1406	1412	-	AAGGCCA
MA1111.2.NR2F2	5.3192377	0.817048208591804	BGNpromoter	106	112	-	GGGGTCA
MA1111.2.NR2F2	5.3192377	0.817048208591804	BGNpromoter	114	120	+	GGGGTCA

Table S9. Blood biochemical indicators testes of tumor-bearing mice treated with DMSO and CIA1.

Testing items	DMSO	CIA1	Reference range	Units
ALT	41.436 ± 2.489	33.836 ± 15.866	10.06-96.47	U/L
AST	127.397 ± 18.218	112.249 ± 35.953	36.31-235.48	U/L
ALB	32.594 ± 2.068	30.207 ± 4.962	21.22-39.15	g/L
ALP	185.351 ± 22.595	208.753 ± 48.028	22.52-474.35	U/L
γ-GT	0.870 ± 0.267	0.833 ± 0.186	0-7.78	U/L
TBIL	13.457 ± 4.301	13.577 ± 6.132	6.09-53.06	μmol/L
DBIL	3.216 ± 0.444	2.478 ± 0.209	0.45-33.89	μmol/L
BUN	2.436 ± 1.420	2.332 ± 0.074	10.81-34.74	mg/dL
CREA	16.107 ± 5.332	14.335 ± 5.036	10.91-85.09	μmol/L
UA	45.447 ± 0.782	60.445 ± 12.120	44.42-224.77	μmol/L

Table S10. Blood routine testes of tumor-bearing mice treated with DMSO and CIA1.

Testing items	DMSO	CIA1	Reference range	Units
WBC	9.8 ± 1.90	7.1 ± 2.86	0.8-10.6	10^9/L
Lymph#	6.0 ± 1.83	4.7 ± 2.52	0.6-8.9	10^9/L
Mon#	0.4 ± 0.32	0.3 ± 0.16	0.04-1.4	10^9/L
Gran#	3.4 ± 0.16	2.1 ± 1.10	0.23-3.6	10^9/L
Lymph%	60.23 ± 7.27	64.80 ± 11.99	40-92	%
Mon%	3.93 ± 2.96	5.08 ± 4.06	0.9-18	%
Gran%	35.84 ± 7.42	30.13 ± 12.53	6.5-50	%
RBC	10.53 ± 0.83	10.7 ± 0.80	6.5-11.5	10^{12}/L
HGB	121 ± 14.16	115 ± 4.36	110-165	g/L
HCT	51 ± 3.16	51.8 ± 3.27	35-55	%
MCV	48.5 ± 2.87	48.5 ± 4.67	41-55	fL
MCH	15.4 ± 1.64	15.2 ± 1.78	13-18	pg
MCHC	337 ± 19.54	321 ± 6.86	300-360	g/L
RDW	15.3 ± 2.74	16.3 ± 1.10	12-19	%
PLT	1324 ± 54.74	1171 ± 74.55	400-1600	10^9/L
MPV	4.9 ± 0.64	5 ± 0.89	4.0-6.2	fL
PDW	16.2 ± 1.55	16.3 ± 1.01	12.0-17.5	
PCT	0.354 ± 0.25	0.542 ± 0.21	0.100-0.780	%
